# Supplementary material for: Identifying content to improve risk assessment communications within the Risk Profile: Literature reviews and focus groups with expert and non-expert stakeholders
Source: PLoS One. 2022 Apr 11;17(4):e0266800. doi: 10.1371/journal.pone.0266800 (PMC9000125; doi:10.1371/journal.pone.0266800)
Supplement: S1 File — (PDF) [file pone.0266800.s001.pdf]

S1 File. Literature search (short report) – supplement material

Ellermann C, McDowell M, Schirren C, Lindemann AK, Koch S, Lohmann M, Jenny MA.
Identifying content to improve risk assessment communications within a Risk Profile: Literature reviews and Focus groups with expert and non-expert stakeholders

Table of Contents

Figure index ..... *II*

Table index ..... *II*

1. Overview *1*

2. Methods *1*

2.1. Search strategy development **1**

2.2. Search methods for identification of studies **1**

2.3. Screening and selection of studies **2**

2.4. Inclusion/exclusion criteria **2**

2.5. Data extraction **3**

2.6. Assessment of methodological quality **3**

2.7. Data synthesis **3**

3. Results *3*

3.1. Search on 'How to communicate food or health risks' **3**

3.2. Search on 'How to communicate probabilities' **14**

3.3. Search on 'How to communicate uncertainty/quality of evidence' **28**

3.4. Search on 'How to communicate the dose-response relationship/thresholds' **40**

3.5. Search on 'How to communicate the severity of health impairments' **44**

4. References *52*

S1 File. Literature search (short report) – supplement material

Figure index

Figure 1: Study selection flow chart – Food/ health risk communication search ... 4
Figure 2: Study selection flow chart - Probability search ... 16
Figure 3: Study selection flow chart – Quality of evidence/ Uncertainty search ... 29
Figure 4: Study selection flow chart - Dose-response search ... 43
Figure 5: Study selection flow chart - Severity search ... 46

Table index

Table 1: Studies excluded with reasons – Food/ health risk communication search ... 4
Table 2: Summary of study quality ratings – Food/ health risk communication search ... 6
Table 3: Summary of study characteristics - Food/ health risk communication search ... 8
Table 4: Studies excluded with reason - Probability search ... 16
Table 5: Summary of study quality ratings – Probability search ... 17
Table 6: Summary of study characteristics - Probability search ... 19
Table 7: Studies excluded with reason – Quality of evidence/ Uncertainty search ... 29
Table 8: Summary of study quality ratings – Quality o evidence/ Uncertainty search ... 32
Table 9: Summary of study characteristics – Quality of evidence/ Uncertainty search ... 33
Table 10: Studies excluded with reason – Severity search ... 47
Table 11: Summary of study quality ratings – Severity search ... 48
Table 12 Summary of study characteristics - Severity search ... 49

S1 File. Literature search (short report) – supplement material

1. Overview

The supplementary material provides a short yet comprehensive report on the search strategy and results for the rapid reviews conducted to inform the development of risk profile. Rapid reviews were employed to generate an overview of the latest evidence on risk assessment communication topics to support development while ensuring that the scientific imperative of methodological rigor was met.

The purpose of these reviews was not to integrate findings into a single review; rather the searches were independent and focused on identifying communication strategies for each of the individual dimensions (e.g., communicating uncertainty, visualizing thresholds). This is consistent with an evidence-based approach and was also applied in the process of creating the German guideline on evidence-based patient information (Lühnen, Albrecht, Mühlhauser, & Steckelberg, 2017).

Detailed information (e.g., data extraction sheets, study quality sheets) is available from the authors on request.

2. Methods

2.1. Search strategy development

Two researchers (CE and CS) developed the search strategies for the different questions. The development of the search strategy was supported by an information specialist from the library of the Max Planck Institute for Human Development.

In order to gain an impression of the general topic and an overview on risk communication in risk assessment, orientational searches were carried out as a first step. These searches included broader questions (e.g., how should risks be communicated to lay target groups?) and also served to identify relevant keywords for the subsequent systematic search.

In face of the limited time available to conduct extensive systematic literature searches on the current state of research for each individual aspect of the *BfR Risk Profile* and the very broad field of research covered by the topics, a more compact approach was undertaken. Several rapid systematic reviews were conducted from November 2017 to February 2018. At the time we conducted the rapid reviews, there was no uniform method for conducting rapid reviews. Nevertheless, the methodological steps we took adhere to all recommendations outlined in the recently published Cochrane Rapid Reviews Methods group guidelines for conducting rapid reviews (with the single exception that the protocol was not published online; Garritty et al., 2021).

After search terms for each research question were collected in a PICO scheme, inclusion and exclusion criteria were defined.

We took a comprehensive approach to identify and revise relevant search terms. First, we dedicated multiple joint research meetings to identify relevant search terms and further refined these in several meetings with a dedicated information specialist from the library of the Max Planck institute for Human Development. These terms were iteratively adjusted to refine the search strategies multiple times. The final search terms excluded a number of initially promising terms because the hits were completely off topic and drastically increased the proportion of irrelevant studies (these terms were excluded after repeated reviews of search result samples).

Keywords for each question were searched in titles and abstracts. Results were downloaded into the EndNote reference management program (version X8) and duplicates removed.

2.2. Search methods for identification of studies

The systematic literature searches were carried out in the electronic databases PubMed, PsycINFO (via Ovid), Scopus, and Web of Science by using the respective keyword system of each database (e.g., Medical Subject

Headings – MeSH in PubMed) and the free text searches. Terms were linked through the Boolean operators "AND" and "OR".

Owing to the different content within each topic, the use of databases were adjusted to the specific search such that the same set of databases were not used for all key questions. Specifically, for some searches, the use of additional databases were employed to find relevant studies (e.g., the use of educational research databases to search for dose-response relationships).

2.3. Screening and selection of studies

Searches were conducted and results of all included databases were stored in EndNote libraries for each search topic. After duplicates were removed, titles and abstracts were initially screened according to the selection criteria (predefined inclusion and exclusion criteria) by one reviewer. A second reviewer checked 30% of a random sample. Potentially relevant papers were screened for final inclusion by two reviewers. In the event of uncertainty, the studies in question were discussed with a third reviewer. The data extraction process was made transparent by using the PRISMA Flow Chart by Moher et al. 2009 (Moher, Liberati, Tetzlaff, Altman, & Group, 2009).

2.4. Inclusion/exclusion criteria

Types of studies

Of interest for data synthesis were systematic reviews, with or without meta-analyses, and primary studies. Preference was given to systematic reviews, meta-analyses, and primary studies that were randomized controlled trials (RCTs) and quasi-randomized controlled trials, which provide the best quality of evidence to inform decision-making (Gates et al., 2018). Systematic reviews (with or without meta-analyses) were prioritised, with RCTs supporting or supplementing these reviews where they were outdated or where no systematic reviews were available. If the question was not addressed from these study types, additional study designs were reviewed (e.g., controlled before-and-after studies without randomization, observational studies, surveys or qualitative studies). Expert opinions were not included. To focus on the most recent evidence, searches were limited to the 10 years preceding execution of the searches (2007/2008 up until 2017/2018). Any deviations from the inclusion and exclusion criteria were noted for each question.

Types of articles/interventions

Studies were included that evaluated the effectiveness of strategies for communicating the relevant aspect stated in the research question (e.g., quality of evidence). No peer-reviewed journals were excluded to ensure appropriate academic rigor.

Types of participants

The main target group of the studies were people without previous scientific experience, who are also the identified target group of the *BfR Risk Profile* (i.e., consumers).

Types of outcome measures

Outcomes of interest were comprehension, knowledge, decision, acceptance, and attitudes or perceptions.

S1 File. Literature search (short report) – supplement material

2.6. Data extraction

Data extraction was initially performed by one reviewer and double-checked by a second reviewer. Information extracted from studies included authors, objective, methods (study design, sample, intervention, outcomes), results, and conclusion. For systematic reviews, we also extracted the date of last search, the included study designs, and the number of studies. For primary studies, we also extracted the year of study, the study design, and the population size. Data extraction sheets for the included studies are available upon request.

2.7. Assessment of methodological quality

Methodological quality was assessed using the AMSTAR I tool (Shea et al., 2007) for systematic reviews, which was the only sufficiently validated tool for the evaluation of systematic reviews (as of 2018), the NIH quality assessment tool for observational cohort and cross-sectional studies (National Heart & Institute, 2014) and the Cochrane Risk of Bias Tool for RCTs (Higgins et al., 2011). The methodological quality was independently evaluated by two reviewers and discussed in cases where there were inconsistencies. Study quality sheets for the included studies are available in a detailed report on the literature review upon request.

2.8. Data synthesis

Evidence for each search was summarized descriptively. Here we present a brief overview of the main findings from each search.

3. Results

This section contains a brief overview of the results for each of the literature searches.

3.1. Search on 'How to communicate food or health risks'

To gain an overview and general impression of (food or health) risk communication from various areas, we conducted a basic search on "How to communicate food risks or health risks from consumer products" in PubMed and Google Scholar in November 2017.

Search strategy – Food/health risk communication search

**PubMed**
(communicat*) AND (((“food risk” OR “food contamination” OR “chemical hazards” OR “biological hazards”)) OR ((“health risk*”) AND (“consumer products” OR “chemical substances” OR “biological substances” OR “substances”)))
**Google scholar**
Keywords: communication, visualisation, risk, food risk, health risk, consumer products

Changes in inclusion/exclusion criteria

The search was not restricted to a publication year.

Results

The literature search in PubMed revealed a total of 406 results (before deduplication). After duplications were removed automatically in Endnote 402 hits were left. In addition, four publications were identified through

search for gray literature in Google Scholar. A total of 406 publications remained after title and abstract screening. After exclusion of 378 publications, 28 remained for full-text screening (see **Figure 1**). Identification of three systematic reviews led to exclusion of 25 publications, with reasons (see **Table 1**).

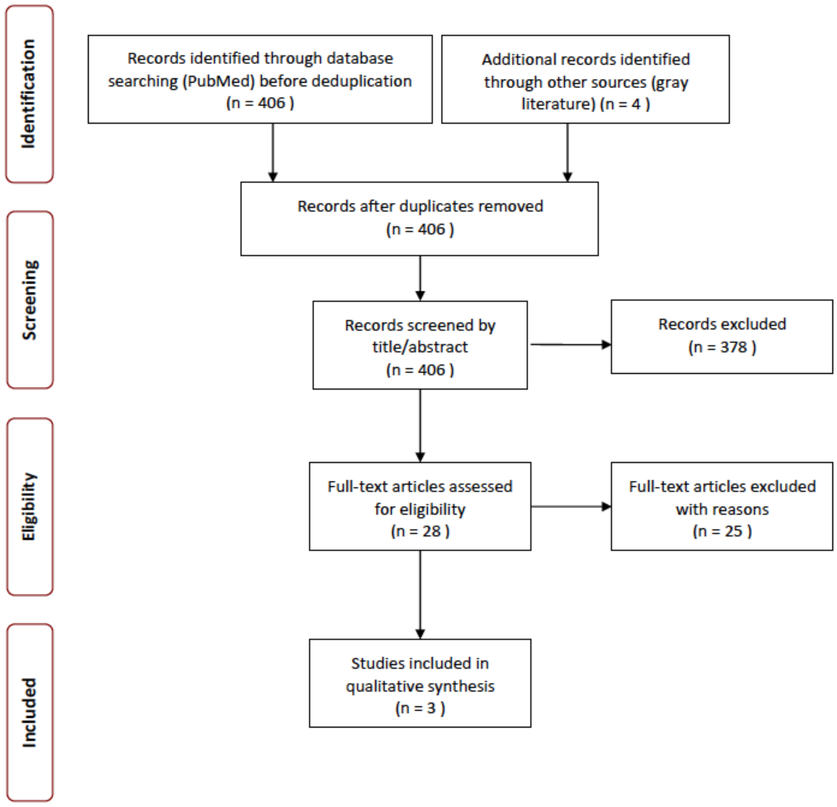

Figure 1: Study selection flow chart – Food/ health risk communication search

Table 1: Studies excluded with reasons – Food/ health risk communication search

	Study	Exclusion reason
1	Assmuth, T. (2011). “Policy and science implications of the framing and qualities of uncertainty in risks: toxic and beneficial fish from the Baltic Sea.” Ambio 40(2): 158-169.	Expert opinion
2	Barnett, J., et al. (2011). “Development of strategies for effective communication of food risks and benefits across Europe: design and conceptual framework of the FoodRisC project.” BMC Public Health 11: 308.	Project development report
3	Connelly, N. A. and B. A. Knuth (1998). “Evaluating risk communication: examining target audience perceptions about four presentation formats for fish consumption health advisory information.” *Risk Anal* 18(5): 649-659.	Non-randomized study
4	Derrick, C. G., Miller, J. S., & Andrews, J. M. (2008). A fish consumption study of anglers in an at-risk community: a community-based participatory approach to risk reduction. *Public Health Nurs*, 25(4), 312-318.	Communication on risk, but not explicit explained how risk should be communicated; before-after trial
5	Fischer, A. R., et al. (2005). “Improving food safety in the domestic environment: the need for a transdisciplinary approach.” *Risk Anal* 25(3): 503-517.	Perspective
6	Hinks, J., et al. (2009). “Views on chemical safety information and influences on chemical disposal behaviour in the UK.” *Sci Total Environ* 407(4): 1299-1306.	Evaluation of semi-structured, focus group
7	Jardine, C. G. (2003). “Development of a public participation and communication protocol for establishing fish consumption advisories.” *Risk Anal* 23(3): 461-471.	Case study report

8	Kaptan, G., et al. (2017). “Extrapolating understanding of food risk perceptions to emerging food safety cases.” *Journal of Risk Research*: 1-23.	Case study analysis
9	Knuth, B. A., et al. (2003). “Weighing health benefit and health risk information when consuming sport-caught fish.” *Risk Anal* 23(6): 1185-1197.	Qualitative study
10	Lee, T. R. (1986). “Effective communication of information about chemical hazards.” *Sci Total Environ* 51: 149-183.	Literature review
11	Lohmann, M., et al. (2013). “[Risk communication of the Federal Institute for Risk Assessment during a food-related outbreak].” *Bundesgesundheitsblatt Gesundheitsforschung Gesundheitsschutz* 56(1): 102-109.	Population survey, non-randomized
12	McCluskey, J. and J. Swinnen (2011). “The media and food-risk perceptions.” *EMBO Rep* 12(7): 624-629.	Literature report
13	McGloin, A., et al. (2009). “Symposium on ‘The challenge of translating nutrition research into public health nutrition’. Session 5: Nutrition communication. The challenge of effective food risk communication.” *Proc Nutr Soc* 68(2): 135-141.	Literature review
14	Murakami, M., et al. (2016). “Evaluation of Risk Perception and Risk-Comparison Information Regarding Dietary Radionuclides after the 2011 Fukushima Nuclear Power Plant Accident.” *PloS One* 11(11): e0165594.	Non-randomized study
15	Nan, X., et al. (2017). “Mapping Sources of Food Safety Information for U.S. Consumers: Findings From a National Survey.” *Health Commun* 32(3): 356-365.	Secondary analysis of survey data
16	Palou, A., et al. (2009). “Integration of risk and benefit analysis-the window of benefit as a new tool?” *Crit Rev Food Sci Nutr* 49(7): 670-680.	Expert opinion
17	Pfau, E. J., et al. (2013). “Communicating local food risk during counterinsurgency operations: development and evaluation of a risk communication campaign.” *US Army Med Dep J*: 51-62.	No fulltext available
18	Renn, O. (2005). “Risk perception and communication: lessons for the food and food packaging industry.” *Food Addit Contam* 22(10): 1061-1071.	Literature review
19	Severtson, D. J., & Vatovec, C. (2012). The theory-based influence of map features on risk beliefs: self-reports of what is seen and understood for maps depicting an environmental health hazard. *J Health Commun*, 17(7), 836-856.	Qualitative study
20	Sillence, E., et al. (2016). “Examining trust factors in online food risk information: The case of unpasteurized or ‘raw’ milk.” *Appetite* 99: 200-210.	Non-randomized study and qualitative interview
21	Tinker, T. L., et al. (2001). “Key challenges and concepts in health risk communication: perspectives of agency practitioners.” *J Public Health Manag Pract* 7(1): 67-75.	Focus group interviews
22	Tinker, T. L. (1996). “Recommendations to improve health risk communication: lessons learned from the U.S. Public Health Service.” *J Health Commun* 1(2): 197-217.	No fulltext available
23	van Dijk, H., Houghton, J., van Kleef, E., van der Lans, I., Rowe, G., & Frewer, L. (2008). Consumer responses to communication about food risk management. *Appetite, 50*(2-3), 340-352. doi:10.1016/j.appet.2007.08.011	Combined FRMQ (consumer perceptions of food risk management quality) score makes no sense.
24	Verbeke, W., et al. (2007). “Why consumers behave as they do with respect to food safety and risk information.” *Anal Chim Acta* 586(1-2): 2-7.	Literature review
25	Williams, P. R. (2004). “Health risk communication using comparative risk analyses.” *J Expo Anal Environ Epidemiol* 14(7): 498-515.	Literature review

The methodological quality of the three included reviews varied (see **Table 2**). Two reviews were of moderate quality, and one was of high quality. None of them performed a meta-analysis. In one review that evaluated the communication of food risks to consumers and/or citizens, in order to identify effective risk communication (Frewer et al., 2016) no comprehensive literature search was performed, and no list of studies (included and excluded) and no characteristics of the included studies were provided. Furthermore, no scientific quality of the included studies was assessed and documented, and therefore could not be used to formulate conclusions. The likelihood of publication bias was not addressed. In another review with two publications that evaluated visualisation techniques for the development of static and dynamic visualisation of risks and benefits (Hallgreen et al., 2016; Mt-Isa et al., 2013) duplicate study selection and data extraction was missing. Characteristics of the

included studies as well as study quality of included studies were not provided. Scientific quality of the included studies was not used in formulating conclusions. The remaining review that evaluated the most effective strategies and techniques for communicating environmental health risks to the public was of high methodical quality (Fitzpatrick-Lewis, Yost, Ciliska, & Krishnaratne, 2010).

Table 2: Summary of study quality ratings – Food/ health risk communication search

	AMSTAR rating											Summary			
Study	**1. Was an " a priori" design provided?**	**2. Was there duplicate study selection and data extraction?**	**3. Was a comprehensive literature search performed?**	**4. Was the status of publication (i.e. grey literature) used as an inclusion criterion?**	**5. Was a list of studies (included and excluded) provided?**	**6. Were the characteristics of the included studies provided?**	**7. Was the scientific quality of the included studies assessed and documented?**	**8. Was the scientific quality of the included studies used appropriately in formulating**	**9. Were the methods used to combine the findings of studies appropriate?**	**10. Was the likelihood of publication bias assessed?**	**11. Was the conflict of interest stated?**	**Yes (y)**	**No (n)**	**Can`t answer (ca)**	**Not applicable (na)**
**Fitzpatrick-Lewis et al. 2010**	y	y	y	y	y	y	y	y	na	n	y	9	1	0	1
**Frewer et al. 2016**	y	y	n	y	n	n	n	n	na	n	y	4	6	0	1
**Mt-Isa et al. 2013, Hallgreen et al. 2016**	y	n	y	y	y	n	n	na	na	na	y	5	3	0	3
Score: 8 to 11 = high quality, 4 to 7 = moderate quality, < 4 = low quality															

Characteristics of the included systematic reviews are summarized in short in Table 3.

Summary of findings to inform risk profile development

In summary, on the basis of the review "How to communicate food risks or health risks from consumer products" we made the following suggestions for the revision of the *BfR Risk Profile*:

- Target groups should be involved in the development of health-related information and the provision of information should be adapted to their needs.
- Information should be complete and well structured (e.g., using question-answer formats, presenting the information in both text and in table format, and including uncertainty information).
- To strengthen confidence in communication, all parties involved should be informed in a timely, consistent, easily understandable, and trustworthy manner.
- In order to prevent extreme avoidance behavior, not only risks but also benefits should be communicated.
- Concrete recommendations for behavioral changes should be given that are feasible for consumers to implement.

- Visuals must be adapted to the needs of the intended audience and the main message that the visuals are intended to convey and also tested for transferability to the *BfR Risk Profile.*

S1 File. Literature search (short report) – supplement material

Table 3: Summary of study characteristics - Food/ health risk communication search

Study, year	Objective	Study design	Search	Studies and sample	Intervention	Outcomes	Authors` conclusion
Fitzpatrick-Lewis et al. 2010	Evaluation on most effective strategies and techniques for communicating environmental health risks to the public.	Systematic Review	Included journals were searched for relevant publications from the date of their inception to November 30, 2009.	24 publications, including randomized controlled trials, cohort studies, interrupted time series, qualitative studies.<br><br>Total sample size ranged from 80 to 3,546	**Population**:<br>U.S. adult men and women were most common.<br><br>4 studies form the Netherlands, 1 in Canada, 1 in UK.<br><br>**Intervention**:<br>Print information, media approaches were the most frequently used method of risk communication. In one study print information was compared to in-person/verbal communication. One study compared in-person/verbal versus no communication.	Most common outcome was change in knowledge; furthermore, behaviour was measured with both hypothetical and actual behaviour. Few studies measured additional outcomes such as judgement and acceptance of risk communication.	Multimedia approaches with combined information types (e.g., texts and diagrams) that address the needs of the target audience and take place in a personal interaction are proposed as the most effective communication strategy to reach a large audience.
Frewer et al. 2016	Evaluation on communicating food risks as well as benefits and harms to consumers and/or citizens, in order to identify effective risk communication, reduce high-risk consumer behaviour or to provide the basis for	Systematic Review	The search was performed on 5th September 2011 in Scopus.	54 papers (experiments (N = 24), surveys (N = 15), qualitative methods (focus groups and interviews, N = 7). 1 paper reported longitudinal time series analysis. 7 papers reported utilizing two types of methods: both surveys and qualitative	**Population:**<br>In 46% (N = 25) of the paper participants were from the general public. Furthermore, study participants were specifically targeted populations such as pregnant women or those exposed to risk through behavior, e.g., people who engaged in	Most frequent: changing attitudes and perceptions (risk or benefit), opinions, or other potential cognitive determinants of behaviour (N = 38, 67%)	Three broad themes relevant to the development of best practice in risk (benefit) communication were identified: the characteristics of the target population; the contents of the information; and the characteristics of the

	informed decisions on food consumption.			methods and surveys (N = 6), or experiments and qualitative methods combined with experiments (N = 1).	fishing for personal consumption (N = 9, 17%). In 31% (N = 17) of the included papers, participants were drawn from populations broader than those who were the intended recipients of the communication.<br><br>Most papers reported on data originating in Europe (N = 26) or Northern America (N = 23). A few papers reported on data from Asia (N = 4) and Australia (N = 1).<br><br>**Intervention:**<br><br>Most of the papers used one-way communication, only in 3 papers dialogue was reported. Most frequently used methods were leaflets (N = 14), information on packaging labels (N = 3); internet (N = 1). Several papers included the use of multiple channels: video or audio in combination with leaflets (N = 5), television and newspapers (N = 2); internet and leaflets (N = 1); and verbal		information sources. Within these themes, independent and dependent variables differed considerably. Overall, acute risk (benefit) communication will require advances in communication process whereas chronic communication needs to identify audience requirements. Both citizen's risk/benefit perceptions and (if relevant) related behaviours need to be taken into account, and recommendations for behavioural change need to be concrete and actionable.

					presentation and leaflets (N = 1).		
Mt-Isa et al. 2013, Hallgreen et al. 2016	Evaluation on visualisation techniques for the development of static and dynamic visualisation of risks and benefits.	Systematic review	Systematic searches were performed for the period after year 2000 on Scopus, PubMed, Web of Knowledge and PsycINFO.	36 papers presenting visuals for benefit-risk communication.	**Population**: N.A.<br><br>**Intervention:**<br><br>Benefit-risk visualisation for different stakeholders in different situations.	Vision perception and comprehension.	1) Prior to the generation of visuals the visual capability of the target population should be evaluated to ensure that the visuals are suitable for the target population.<br>2) The Wicken's Principles of Display Design should be considered when creating visualisations.<br>3) The GlaxoSmithKline (GSK) Graphics Principles should be used when creating graphics.<br>4) Planning Stage: When structuring a decision problem, a tree diagram should be created to indicate the hierarchy, and a table with the required data should be prepared.<br>5) Evidence gathering and data preparation stage: The table prepared in step 4 should be completed while gathering data. The tree diagram should be discussed anew. Risk ladders or pictograms/icons are

							useful for the presentation of the data to the general public.<br>6) Analysis stage: Benefit-risk magnitudes and the stakeholder’s value preferences should be represented by bar graphs, dot plots or line graphs.<br>7) Exploration stage: After results are verified for robustness, the following tools should be used in this stage: line graphs, distribution plots and forest plots<br>8) Bar graphs should be used for comparison of magnitudes oft the final benefit-risk metrics.<br>9) Only tables, risk scales/ladders, pictograms, pictographs and icon arrays should be used to compare quantitative data like probabilities,<br>10) Only line graphs, dot plots and forest plots should be used to provide an easily

							understandable visual for changes of one variable against another one.<br>11) Only distribution plot, box plot, forest plot and tornado diagram are useful to visualize the distributions or uncertainties of a benefit-risk metric.<br>12) The contributions of different criteria in the benefit-risk-analysis should only be visualised using bar graphs.<br>13) The strength of relationships should be visualised by scatter plots or tornado diagrams.<br>14) The different statistical significances of alternatives should be visualised in a distribution or forest plot.<br>15) For qualitative data only table, tree diagram, pictogram or cartoons/icons should be used.<br>16) Categorial data should be visualised using bar graphs or dot plots.

							17) The use of interactive displays allows an interactive exploration of benefit-risk models.

3.2. Search on 'How to communicate probabilities'

In the literature search on "How to communicate probabilities", we defined probabilities as risks with a known probability of occurrence. The literature search was conducted from 01-12-2017 to 08-12-2017 in four predefined databases (PubMed, PsycINFO, Web of Science, Scopus).

Search strategy – Probability search

**PubMed**			
**communication**	**probability**	**risks**	**outcome**
((“Communication”[Mesh] OR communicat*) AND (Visual* OR Verbal* OR Numeric* OR Graphic* OR Format OR Image OR “risk ladder*” OR “decision aid*” OR Label* OR Analogy OR Analogies))	“Probability”[Mesh] OR “statistics and numerical data”[Subheading] OR number* OR “Odds Ratio”[Mesh] OR “Uncertainty”[Mesh] OR ambig* OR “Risk”[Mesh] OR Certaint* OR “expert opinion”	Hazard* OR Risk OR Uncertaint* OR Benefit* OR Harms OR harmful OR Certaint* OR vague	“Education”[Mesh] OR “Patient Medication Knowledge”[Mesh] OR “Knowledge”[Mesh] OR “Health Knowledge, Attitudes, Practice”[Mesh] OR “Knowledge of Results (Psychology)”[Mesh] OR “Perception”[Mesh] OR “Comprehension”[Mesh] OR Understand* OR Comprehension* OR “risk perception” OR Decision* OR “informed decision” OR “informed choice” OR “Decision Making”[Mesh]
MA SR, last 5 y: 161			
**PsycINFO**			
**communication**	**probability**	**risks**	**outcome**
(Communicat*).ti,ab. AND (Visual* OR Verbal* OR Numeric* OR Graphic* OR Format OR Image OR “risk ladder*” OR “decision aid*” OR Label* OR Analogy OR Analogies).ti,ab.	(Probabilit* OR Likelihood* OR Numbers OR Risk* OR Uncertain* OR Certaint* OR “expert opinion”).ti,ab.	(Hazard* OR Risk OR Uncertaint* OR Benefit* OR Harm* OR Certaint* OR vague).ti,ab.	(Understand* OR Comprehen* OR Interpretation* OR “perceived risk” OR “risk perception” OR “estimated risk” OR “risk 14andomiz*” OR “informed choice*” OR Decision* OR Preference*).ti,ab.
MA SR, last 5 y: 65			
**Web of Sicence**			
**communication**	**probability**	**risks**	**outcome**

S1 File. Literature search (short report) – supplement material

#1 TS=("communication" OR communicat*) OR<br><br>#2 TS=((visual* or verbal* or numeric* or graphic* OR format OR image OR "risk ladder*" OR "decision aid*" OR label* OR analogy OR analogies))<br><br>#3 #1 OR #2	#4 TS=(probability OR probabilities OR number* OR "Odds Ratio" OR Uncertainty OR ambig* OR Risk* OR Certaint* OR "expert opinion")	#5 TS=(Hazard* OR Risk OR Uncertaint* OR Benefit* OR Harm* OR Certaint* OR vague)	#6 TS=(Understand* OR Comprehension* OR "risk perception" OR Decision* OR "informed decision" OR "informed choice")<br><br>#3 AND #4 AND #5 AND #6
Indexes=SCI-EXPANDED, SSCI, A&HCI, CPCI-S, CPCI-SSH, BKCI-S, BKCI-SSH, ESCI, CCR-EXPANDED, IC Timespan=2012-2017			
MA SR, last 5 y: 76			
**Scopus**			
**communication**	**probability**	**risks**	**outcome**
TITLE-ABS-KEY (communication OR communicate) AND TITLE-ABS (visual* OR verbal* OR numeric* OR graphic* OR format OR image OR "risk ladder*" OR "decision aid*" OR label* OR analogy OR analogies)	TITLE-ABS-KEY<br><br>(probability OR probabilities OR ambig* OR risk* OR certaint* OR "expert opinion")	TITLE-ABS-KEY (hazard* OR risk OR uncertaint* OR benefit* OR harm* OR certaint* O R vague)	TITLE-ABS-KEY<br><br>(understand* OR compre hension* OR "risk perception" OR decision* OR "informed decision" OR "informed choice")<br><br>AND PUBYEAR > 2012 AND PUBYEAR < 2018
MA SR, last 5 y: 93 (TITLE-ABS-KEY ("Systematic Review" OR "Metaanalys*" OR "Meta-analys*"))			

Changes in inclusion/exclusion criteria

Because this topic is very well researched, we solely included systematic reviews and meta-analyses published within the five years prior to our search (December 2012 to December 2017).

Results

The systematic literature search revealed a total of 396 hits: 161 from PubMed, 66 from PsycINFO (EBSCOhost), 76 from Web of Science, and 93 from Scopus. After removal of duplicates, 335 publications were left. After title and abstract screening, 11 publications were left for full-text screening. After exclusion of two publications with reason (see **Table 4**) nine systematic reviews were included for data synthesis (see **Figure 2**).

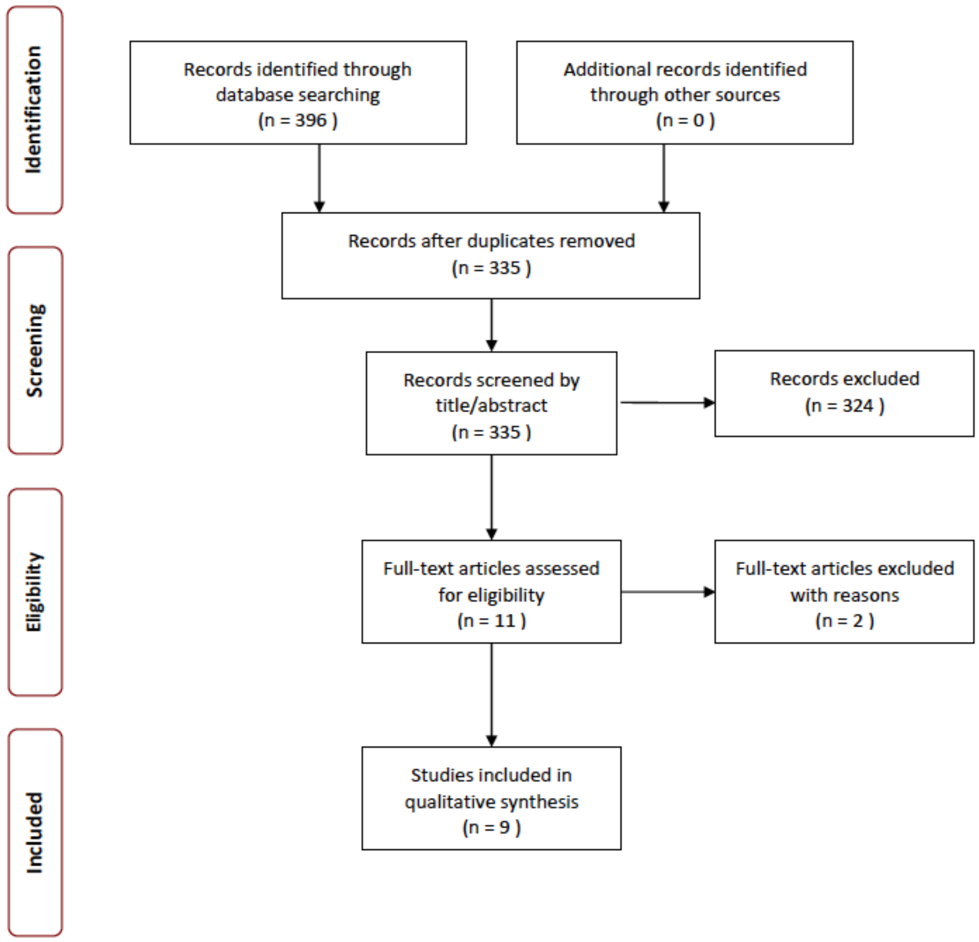

Figure 2: Study selection flow chart - Probability search

Table 4: Studies excluded with reason - Probability search

	**Study**	**Exclusion reason**
1	Dhami et al. 2015. Improving intelligence analysis with decision science.	No systematic review or literature review, just perspective
2	Garcia-Retamero & Cokely 2013. Communicating Health Risks With Visual Aids.	More recent and systematic paper by the same authors available

The methodological quality of the included systematic reviews varied from moderate (Barros et al. (2014); Garcia-Retamero & Cokely, 2017; Harrison et al., 2014) to high (Büchter, Fechtelpeter, Knelangen, Ehrlich, & Waltering, 2014; French, Cameron, Benton, Deaton, & Harvie, 2017; Lühnen et al., 2017; Stellamanns, Ruetters, Dahal, Schillmoeller, & Huebner, 2017; West et al., 2013; Zipkin et al., 2014) (see **Table 5**). In three reviews (Barros et al., 2014; Garcia-Retamero & Cokely, 2017; Harrison et al., 2014) study quality was not assessed and was therefore not adequately considered in the formulation of the conclusions. Furthermore, conflict of interest was not stated in one of the three reviews (Barros et al., 2014). In one of the three reviews the status of publication (i.e. grey literature) was not used as an inclusion criterion (Harrison et al., 2014). In another systematic review duplicate study selection and data extraction was not clear (Garcia-Retamero & Cokely, 2017). One review did not provide characteristics of the included studies (Harrison et al., 2014) and one did not perform a comprehensive literature search (West et al., 2013). Only one systematic review provided a meta-analysis and therefore achieved the highest AMSTAR score (Büchter, Fechtelpeter, Knelangen, Ehrlich, & Waltering, 2014).

Table 5: Summary of study quality ratings – Probability search

	AMSTAR rating											Summary			
Study	1. Was an " priori" design provided?	2. Was there duplicate study selection and data extraction?	3. Was a comprehensive literature search performed?	4. Was the status of publication (i.e. grey literature) used as an inclusion criterion?	5. Was a list of studies (included and excluded) provided?	6. Were the characteristics of the included studies provided?	7. Was the scientific quality of the included studies assessed and documented?	8. Was the scientific quality of the included studies used appropriately in formulating conclusions?	9. Were the methods used to combine the findings of studies appropriate?	10. Was the likelihood of publication bias assessed?	11. Was the conflict of interest stated?	Yes (y)	No (n)	Can`t answer (ca)	Not applicable (na)
**Barros et al. 2014**	y	y	y	y	y	y	n	n	na	na	n	6	3	0	2
**Büchter et al. 2014**	y	y	y	y	y	y	y	y	y	y	y	11	0	0	0
**French et al. 2017**	y	y	y	y	y	y	y	y	na	na	y	9	0	0	2
**Garcia-Retamero et al. 2017**	y	ca	y	y	y	y	n	n	na	na	y	6	2	1	2
**Harrison et al. 2014**	y	y	y	n	y	n	n	n	na	na	y	5	4	0	2
**Lühnen et al. 2017**	y	y	y	y	y	y	y	y	na	y	y	10	0	0	1
**Stellamans et al. 2017**	Y	Y	Y	Y	Y	Y	Y	Y	na	Y	Y	10	0	0	1
**West et al. 2013**	y	y	n	y	y	y	y	y	na	na	y	8	1	0	2
**Zipkin et al. 2014**	y	y	y	y	y	y	y	y	na	na	y	9	0	0	2
Score: 8 to 11 = high quality, 4 to 7 = moderate quality, < 4 = low quality															

It should be noted that some primary studies were reported in several reviews. Because of the narrative and not quantitative synthesis for this report, we retained all of the identified reviews.

Characteristics of the included studies are summarized in short in Table 6.

Summary of findings to inform risk profile development

In summary, on the basis of the descriptive review we can make the following suggestions on how to communicate probabilistic information in the *BfR Risk Profile*:

- Risks should be quantified or categorized where possible and numbers used to communicate the magnitude of risks. Verbal labels are interpreted inconsistently and should be avoided.
- When using numbers, frequencies or percentages are most understandable, and the reference class should be made clear (e.g., both the numerator and denominator; detail about to whom or to what the numbers refer).
- If visuals are used, icon arrays appear to be an effective format for communicating risk magnitudes. However, they may not fit within the structure of the *BfR Risk Profile*.
- In order to ensure readability for less educated groups, general recommendations for the layout, structure, and design of texts should be taken into account (e.g., high readability index, clear structure of texts, use of examples, color design of texts).

S1 File. Literature search (short report) – supplement material

Table 6: Summary of study characteristics - Probability search

Study, year	Objective	Study design	Search	Studies and sample	Intervention	Outcomes	Authors` conclusion
Barros et al. 2014	Impact of pictograms on comprehension and compliance of patients.	Systematic Review	Searches were conducted from February to March 2012 in EBSCO, Embase, LILACS, Pubmed, Scopus, SciELO and PsycINFO	Inclusion of 24 studies, of which 50% were conducted in Africa.	**Population**: Patients from hospitals, out-patient facilities and health units. 4 studies form the Netherlands, 1 in Canada, 1 in UK.<br><br>**Intervention**: In 23 studies, pictograms were used to increase understanding and adherence with pharmacotherapy. In one study, they were used to explain prognostic and diagnostic tests.	Correct use of medication (improved effectiveness, reduced risks), adherence	Pictograms can be useful to improve understanding and adherence to pharmaceutic treatments, but they have to be validated carefully concerning their transparency and translucency. If they don't perform well, they may cause confusion and harm. Perception and interpretation of pictograms depends on several factors of the target population, such as culture, level of education, and experience.
Büchter et al. 2014	Effectiveness evaluation of words versus numbers in communicating the probability of adverse effects to consumers in written health information.	Systematic review and meta-analysis	Nine electronic databases were searched up to November 2012.	Identification of 7 publications, including 10 randomised controlled trials.	**Population**: Study participants were recruited from the general population or via a cancer website and confronted with a hypothetical scenario.<br><br>**Intervention:** Participants received short information leaflets on drugs for a particular condition which only differed in whether the	The studies included five outcomes: estimation of probabilities (in percentages), likelihood of occurrence, satisfaction, intention to take or continue to take the medicine and the impact of the information on the decision. All outcomes were measured shortly	Adverse treatment effects presented numerically led to better estimates of risks and increased satisfaction and likelihood of medication use. Further research should consider personal and contextual factors, including the setting,

					information on the frequency of the adverse effects of the drug were presented verbally or numerically. One study examined a combination of a verbal and numerical description, as it is currently included in the 2009 European Commission Guideline on the readability of package leaflets.	after distribution of the information leaflets, and none of the studies had a follow-up.	disease, numeracy and educational.
French et al. 2017	Overview of the effect of changes in health-related behaviour due to personalised risk information, compared to not personalized risk information.<br>Furthermore, the review aims to assess the variation of changing behaviour due to the type of information provided, the type of health-related behaviour targeted by the intervention, and the disease associated with the risky behaviour.<br>In addition, the study aims to summarize the importance of self-efficacy and response efficacy in	Systematic review of systematic reviews of RCT and non-RCTs	Medline, Cochrane Database of Systematic Reviews, CINAHL Plus and PsycInfo were searched in January 2016.	9 systematic reviews, published from 2008 onwards, covering 36 studies.	**Population:**<br>Participants at risk of a behaviour-related disease. They were recruited from the general population or from primary prevention patients.<br>**Intervention:**	Changes in the following health-related behaviours: smoking, alcohol consumption, diet, and physical activity.	There is little evidence that the communication of personalised risk information has long-term effects on health-related behaviour.<br>The most promising methods are imaging/visual approaches, but also with inconsistent results.<br>Successful behaviour-changing interventions should involve behaviour changing techniques that increase the capability of self-regulation, and aim at self-efficacy and response efficacy.

	interventions, behaviour change techniques, and the use of theory to inform and develop interventions.						
Garcia-Retamero & Cokely 2017	Effectiveness evaluation of numeracy on risk literacy, decision making, and health outcomes, and to evaluate the benefits of visual aids in risk communication	Systematic Review	Scientific research published between January 1995 and April 2016;<br>Databases: Web of Science, PubMed, PsycINFO, ERIC, Medline, and Google Scholar	36 publications were included	**Population:**<br>Included Studies involved a total of 27,885 participants from 60 different countries, aged 19-94 (average 45.02). 55,8 of the participants were male. 29% had a High School degree or less education, 34% had some college education, 37% a bachelor or higher degree.<br>Participants were recruited from the general population at state or community level (19%), via online panels (31%), from patient or high-risk populations (22%), from young adults with a high level of education(25%), practising doctors (6%), and from elderly with limited skills (3%).<br>**Intervention:**<br>83% of the studies presented static visual aids and measured their impact on risk literacy and health-related behaviour.	Visuals vs. numbers, visuals vs. other visuals, dynamic vs. static visuals, comprehension, recall, behaviour, risk literacy, numeracy, graph literacy etc.	Visuals work in comparison to no visuals, the type of visual depends on the type of data that is communicated, there is more research in static visuals than dynamic ones, especially icon-arrays have been researched quite well and seem to work, especially for low-skilled individuals. For dynamic visuals, results are mixed.

					65% of these studies compared their results with those of a control group, which got written information without static visual aids. 17% of the studies tested dynamic visual aids in people with different levels of numeracy.		
Harrison et al. 2014	Evaluation of the current methodology of risk communication in healthcare-related discrete choice experiments (DCE), and elaboration of recommendations for improving risk communication in healthcare DCEs	Systematic and narrative review	The Web of Science, MEDLINE, EMBASE, PsycINFO and Econlit databases were searched on 18 April 2013 for DCEs that included a risk attribute published since 1995, and on 23 April 2013 to identify studies assessing risk communication in the general (non-DCE) health literature	**Systematic review of DGE-studies:** 117 peer-reviewed articles published from 2000 onwards were included.<br><br>**Narrative review of risk communication tools:** 99 studies included	**Population:** Participants were patients, carers of patients, parents or healthcare professionals. Participants' literacy and numeracy skills were not assessed.<br><br>**Intervention*:*** Two reviews were performed. First a systematic review of studies using DCEs in which risk is included as attribute to value a health care intervention. Second a narrative review to detect examples for good practice in health communication in non-DCE-studies.<br><br>**Systematic review of DGE-studies:** All studies used discrete choice experiments (DCE) with a healthcare related risk	DCE-Studies: Outcome was decision making, measured by precision, consistency and variability of the DCE-results. 59 (50%) studies also measured the willingness to pay (WTP) for risk reduction. 2 studies have calculated the value of statistical life.<br><br>Review of risk communication tools: Of particular interest was the effectiveness of the presentation tools measured by using numeracy tests, non-numerical questionnaires or interviews.	The literacy of the target population should be assessed.<br><br>To avoid framing bias, positive and negative outcomes of an intervention should be presented. A consistent denominator should be used. Risk should be defined and put into context.<br><br>Qualitative descriptions of risk should be avoided.<br><br>RRR may appear larger than absolute risks. Thus people are more likely to give consent to an intervention. Therefore RRR should only be reported in addition to ARR to provide a complete picture of the risk and to avoid overestimation. The use of visual aids should be considered.

					attribute with or without an opt-out option. Main healthcare topic was cancer (27; 23%). Most studies investigated preferences for pharmaceutical therapy (70; 60%), and screening/diagnostics (15; 13%).<br><br>72 studies presented quantitative information in terms of frequencies, percentages or a combination of both. 18 studies used varying denominators. 19 studies used qualitative information. 26 studies combined quantitative and qualitative information.<br><br>85 studies presented absolute risks, 8 studies presented relative risks, and 5 studies combined both.<br><br>Visual aids were used in 27 studies, mainly risk icons and dot arrays, furthermore bar charts, risk ladders, pictograms, and photographs.<br><br>Most studies (109) used negative framing		The optimal format of risk communication depends from the context (e.g. ex-ante, ex-post). It may be beneficial to involve methodologists from other disciplines to design it.<br><br>Limited evidence was found for the use of background information and training to increase risk numeracy.

					of the risks of an intervention.<br><br>**Narrative review of risk communication tools:** empirical studies, literature overviews without structured literature search, systematic reviews, and studies which looked at presenting uncertainty. 65 studies used numerical presentation, 34 qualitative descriptions, 29 pictures, and 15 graphical tools.		
Lühnen et al. 2017	Systematic review on the presentation of frequencies in the context of developing a guideline on evidence-based health information.	Systematic review, which included randomised controlled trials on the verbal and visual communication of risks.	PubMed, CENTRAL, PSYNDEX/ PsycINFO, CINAHL were searches in January 2014 and November 2013 for RCTs, Systematic Reviews and Meta-Analysis	15 studies with 3.532 participants were included	**Population:**<br>The studies were conducted in the USA, UK, Canada, Australia and Singapore. Pregnant women and mothers, students, patients, citizens and caregivers were included.<br><br>**Intervention:**<br>The interventions consisted of scenarios on side effects of antibiotics, analgesics, statins, tamoxifen, and cancer therapies, medical test results, probabilities of certain events in infants,	Outcome parameters were: risk perception, understanding and knowledge, comprehensibility and readability, acceptance, attractiveness and trustworthiness, and Intention to take the medication.	Information should not only be provided verbally. Numerical information in addition to verbal information increase knowledge and willingness to consent.<br><br>Additional findings of the guideline:<br><br>Graphical aids increase acceptance, while there is little evidence of their impact on risk perception, knowledge and understanding. Narratives should be avoided. Fact boxes increase risk perception, knowledge, readability, and comprehensibility. It's

					stroke risk, and a fictional doctor-family meeting.		recommended to involve laypersons in the development of health information.
Stellamanns et al. 2017	Summary of evidence on computer-supported graphs that present risk data and their effects on various measures	Systematic Review	The search was carried out in August 2015. Literature databases were investigated via EBSCO and OVID search hosts. The EBSCO search included the following databases: Health Source: Nursing/Aca-demic Edition, Library, Information Science & Technology Abstracts, MEDLINE, Psychology and Behavioral Sciences Collection, PsycINFO, CINAHL and ERIC. The EMBASE database was searched via OVID. Additionally, the IEEE Xplore Digital Library was investigated.	13 studies until August 2015, all controlled designs, 10 with static graphs, 3 with dynamic ones, most decision scenarios were hypothetical, all visualisations were computer-based. One study was conducted with actual cancer patients and one with women with a high risk of developing cancer, the rest with lay people.	**Population:** Participants came from the United States, Canada, Australia, New Zealand and Great Britain. Most of them were recruited via websites or online panels. Participants were university students, mothers, women at elevated risk of breast cancer, cancer patients, or were recruited from the general population. **Intervention:** Most studies looked at icon-arrays, mostly in a 10x10 grid, and compared the performance of visuals vs. text-based information. Only few studies compared different types of visuals.	Outcomes of most studies was the intention to take action or behaviour and accurate estimation or reproduction of a numerical value. Some studies measured comprehension, credibility of information, uncertainty, and time to complete a certain task.	Well-designed static graphs can improve web-based cancer risk communication in particular populations. Dynamic formats cannot (yet) be recommended.
West et al. 2013	A literature research to investigate whether the numeric and non-numeric presentation of quantitative risk and benefit information in promotional prescription drug	Systematic Review	PubMed database was searched for articles published between January 1, 1990, and February 23, 2011	52 met the inclusion criteria, of which 37 studies focused on prescription or hypothetical drugs	**Population:** Populations studied were adults of diverse background, including university students, professionals, parents, patients, and adults of	Accuracy of knowledge, understanding, perceived risks and benefits, and, to a lesser extent, behaviour and decision making	Numerical presentation of benefit or risk increased the knowledge gain in participants compared with non-numerical presentation. There was no particular format consistently

	labeling or print advertising has an effect on people's processing, understanding, and behaviour.				the general population.<br>**Intervention:**<br>Numeric and non-numeric presentation of quantitative risk and benefit information in promotional prescription drug labeling or print advertising		superior. The combination of numeric and non-numeric presentation may be useful.<br>Results differed depending on health literacy and numeracy skills of the participants.
**Zipkin et al. 2014**	Effectiveness evaluation of methods used to communicate probabilistic information about harms and benefits.	Systematic Review	PubMed (1966 to March 2014), CINAHL, EMBASE, and the Cochrane Central Register of Controlled Trials (1966 to December 2011) were searched.	84 articles, representing 91 studies. 74 were RCTs, mostly with cross-sectional design. 49 studies were paper-based, and 39 computer-based. 33 studies included patients at risk for a specific condition. 77 studies presented probabilistic data about benefits of the intervention, 21 on harms of the intervention, 7 on benefits and harms.	**Population:**<br>36,3% of the trials included persons at risk of a specific disease. In one study, 50% of the participants were cancer survivors. Participants of 29,7% of the studies were recruited in healthcare settings. Not all authors of the primary studies reported the characteristics of their participants. Of those who did, the median age was 46,17 years. Most participants had some college education (Median= 36%) or a college degree (Median = 35%). The other participants had a high school degree (Median = 27,8%) or less	Cognitive outcomes (not consistently defined in the included studies); affective outcomes: satisfaction, perceived helpfulness, preference for the method, level of decisional conflict, level of worry; behavioural outcome: choice of treatment, willingness to consent of an intervention, acceptance of a procedure.	Cognitive outcomes are improved by the use of visual aids like bar graphs and icon arrays, which include both, affected persons and total population.<br>Natural frequencies may be clearer than event rates, but more research is needed.<br>Absolute differences in risks are more intuitive and result in better accuracy compared to RRR, but are less likely to influence the willingness to consent to intervention. ARRs with additional graphical aids may lead to similar effects of decision making like RRRs.<br>NNTs are less understandable for patients and should be avoided.

					education (Median = 9,4%).<br><br>**Intervention:**<br>Prevailing comparisons: variations of icon array presentations, icon arrays versus bar graphs, ARR versus RRR, event rates versus natural frequencies, NNT versus ARR, NNT versus RRR, natural frequencies versus NNT, RRR versus event rates, RRR versus natural frequencies.		

3.3. Search on 'How to communicate uncertainty/quality of evidence'

We defined uncertainty as quality of the underlying evidence (epistemic uncertainty). To access the most recent evidence on that topic, different approaches were taken. First, we contacted different organisations or experts whom we knew were working on this topic: David Spiegelhalter and Anne Marthe van der Bles from the Winton Centre for Risk and Evidence Communication; the European Food Safety Authority (EFSA), who recently conducted a review on the topic ('Guidance on Communication of Uncertainty in Scientific Assessment')(EFSA (European Food Safety Authority) et al., 2019); and the Institute for Quality and Efficiency in Health Care (IQWiG), which is also working on guidelines for communicating uncertainty in health information.

Having known that two reviews on the communication of uncertainty would soon be published (EFSA (European Food Safety Authority) et al., 2019; van der Bles et al., 2019), we conducted two smaller searches for this question: one cited reference search via Google Scholar on 8-7-2018 on Schünemann et al. (Schünemann, Best, Vist, Oxman, & Group, 2003), a publication that specifically discussed the importance of communicating quality of evidence, and a search for keywords related to the concept of uncertainty/quality of evidence within our results for the probability question, seeing as probability and uncertainty are frequently used interchangeably. In addition, gray literature was collected.

Search strategy - Quality of evidence/uncertainty search

**Search in probability results**
**Keywords**: Uncertain, Ambig, Trustworth, "Quality of evidence", "Evidence quality", Reliab, Validity
**Google scholar**
**Keywords**: Uncertain, Ambig, Trustworth, "Quality of evidence", "Evidence quality", Reliab, Validity

Changes in inclusion/exclusion criteria

We included studies that looked at the effect of communicating quality of evidence either in different formats, such as text, visuals, or numbers, or compared with no communication of evidence quality. Not having expected any evidence based on experiments and in order to gain a general overview of the current literature on this topic, we did not limit the search to study designs.

We excluded studies that focused on the communication of ranges (e.g. confidence intervals) or compared formats that were irrelevant for the *BfR Risk Profile* (for example, studies that looked at the effect on trust depending on who mentions (e.g., physicians or politicians) uncertainty about a medical finding).

Results

The search revealed a total of 55 potentially relevant studies: 12 records identified through repeated search in titles and abstracts of the probability search, 26 records thorough gray literature search, and 17 records identified through cited reference search. After removal of duplicates, 53 publications were left for full-text screening. After exclusion of 46 publications with reason (see **Table 7**), seven were included for data synthesis (see **Figure 3**).

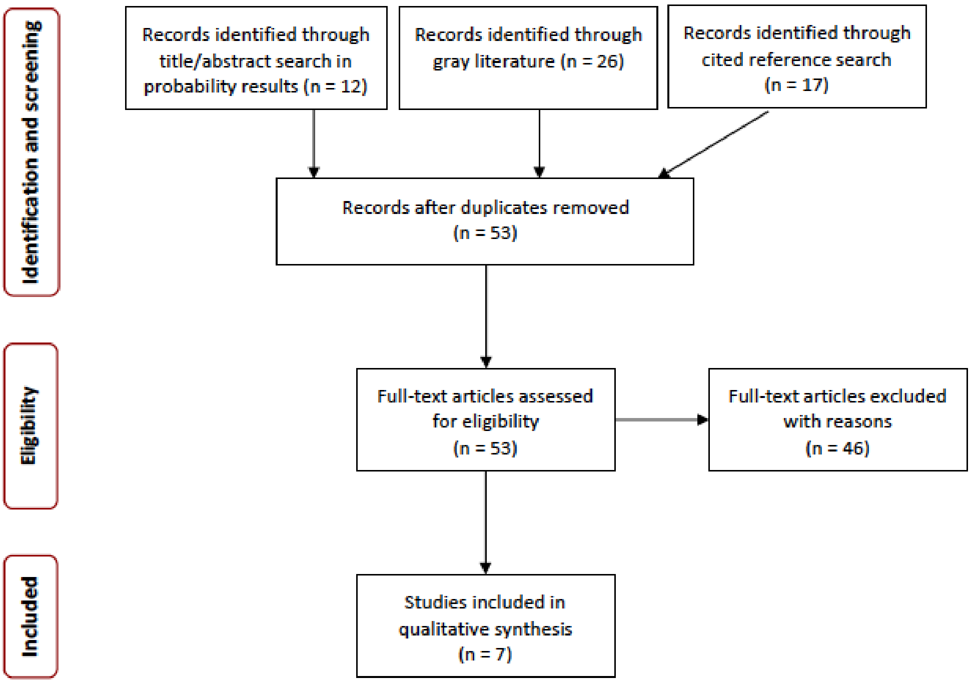

Figure 3: Study selection flow chart – Quality of evidence/ Uncertainty search

Table 7: Studies excluded with reason – Quality of evidence/ Uncertainty search

	Study	Exclusion reason
1	Aguilera-Eguía et al. 2015. Niveles de evidencia y grados de recommendación en kinesionlogía; una comparación entre cinco sistemas de clasificación. *Rev Soc Esp Dolor* 2015, 22(5), 205-211.	Not in English or German
2	Akl et al. 2012. "Might" or "suggest"? No wording approach was clearly superior in conveying the strength of recommendation. *Journal of Clinical Epidemiology,* 65 (2012), 268e275.	Not focussing on questions relevant for us
3	Bansback et al. 2017. Communicating Uncertainty in Benefits and Harms: A Review of Patient Decision Support Interventions. *Patient* (2017) 10, 311.	No experimental results reported
4	Binder et al. 2016. Conflict or Caveats? Effects of Media Protrayals of Scientific Uncertainty on Audience Perceptions of New Technologies. *Risk Analysis,* 36(4), 831-846.	Format tested irrelevant for us
5	Carrasco-Labra et al. 2015. Comparison between the standard and a new alternative format of the Summary-of-Findings tables in Cochrane review users: study protocol for a randomized controlled trial. *Trials*, 16, 164.	No experimental results reported
6	Carrasco-Labra, A., et al. (2016). Improving GRADE evidence tables part 1: a randomized trial shows improved understanding of content in summary of findings tables with a new format. *Journal of clinical epidemiology*, 74, 7-18.	Wrong target group
7	Dhami et al. 2015. Improving Intelligence Analysis With Decision Science. *Perspectives on Psychological Science* 2015, 10(6), 753–757.	Not focussing on questions relevant for us
8	Dieckmann et al. 2012. Making sense of uncertainty: advantages and disadvantages of providing an evaluative structure. *Journal of Risk Research*, 15(7), 717-735.	Not focussing on relevant questions for us (mixing format with ranges)
9	EFSA 2019. Guidance on communication of uncertainty in scientific assessments. *EFSA Journal* 2019, 17(1):5520, 73 pp.	No relevant sources reported
10	Elwyn et al. 2006. Developing a quality criteria framework for patient decision aids: online international Delphi consensus process. *BMJ* 2006, 333, 417.	Not focussing on questions relevant for us
11	Fox et al. 2011. Assimilating evidence quality at a glance using graphic display: research synthesis on labor induction. *Acta Obstet Gynecol Scand* 2012, 91, 885–892.	No experimental results reported

12	Garcia-Retamero et al. 2017. Designing Visual Aids That Promote Risk Literacy: A Systematic Review of Health Research and Evidence-Based Design Heuristics. *Human Factors*, 59(4), 582–627.	Not focussing on questions relevant for us
13	Goerland et al. 2016. On the assessment of uncertainty in risk diagrams. *Safety Science*, 84, 67-77.	No experimental results reported
14	Hall et al. 2017. New Approach to Weight-of-Evidence Assessment of Ecotoxicological Effects in Regulatory Decision-Making. *Integrated Environmental Assessment and Management*, 13(4), 573-579.	No experimental results reported
15	Han 2012. Conceptual, Methodological, and Ethical Problems in Communicating Uncertainty in Clinical Evidence. *Medical care research and review : MCRR, 70*(1 Suppl), 14S-36S.	No experimental results reported
16	Hildon et al. 2012. Impact of format and content of visual display of data on comprehension, choice and preference: a systematic review. *International Journal for Quality in Health Care*, 24,(1), 55–64.	No relevant sources included
17	Hullman et al. 2016. In Pursuit of Error: A Survey of Uncertainty Visualization Evaluation. Retrieved from: https://research.tableau.com/sites/default/files/uncertainty_vis_eval.pdf [29.04.2019].	No experimental results reported
18	Hullmann 2016. Why Evaluating Uncertainty Visualization is Error Prone. Retrieved from: http://users.eecs.northwestern.edu/~jhullman/paper_BELIV_evaluating_uncertainty_vis.pdf [29.04.2019].	No experimental results reported
19	Jensen et al. 2017. Communicating Uncertain Science to the Public: How Amount and Source of Uncertainty Impact Fatalism. *Risk Analysis*, 37, 40-51.	Format tested irrelevant for us
20	Johnson et al. 1995. Presenting Uncertainty in Health Risk Assessment: Initial Studies of Its Effects on Risk Perception and Trust. *Risk Analysis*, 15, 485-494.	Not focussing on questions relevant for us (only ranges)
21	Joslyn et al. 2015. Climate Projections and Uncertainty Communications. *Top Cogn Sci*, 8, 222-241.	Not focussing on questions relevant for us
22	Kaiser et al. 2010. A Meta Schema for Evidence Information in Clinical Practice Guidelines as a Basis for Decision-Making. *Studies in health technology and informatics*, 129(Pt 2), 925-929.	No experimental results reported
23	Khan et al. 2011. Making GRADE accessible: a proposal for graphic display of evidence quality assessments. *Evidence-Based Medicine*, *16*(3), 65-69.	No experimental results reported
24	Kinkeldey et al. 2015. Evaluating the effect of visually represented geodata uncertainty on decision-making: systematic review, lessons learned, and recommendations. *Cartography and Geographic Information Science, 44*(1), 1-21.	Not focussing on questions relevant for us
25	Kristiansen et al. 2014. Development of a Novel, Multilayered Presentation Format for Clinical Practice Guidelines. *Chest, 147*(3), 754-763.	No experimental results reported, wrong target group
26	Lomotan et al. 2010. How “Should” We Write Guideline Recommendations? Interpretation of Deontic Terminology in Clinical Practice Guidelines: Survey of the Health Services Community. *Quality & safety in health care, 19*(6), 509-513.	Not focussing on questions relevant for us
27	Mignini, L., et al. (2016). Graphical displays for effective reporting of evidence quality tables in research syntheses. *Reproductive health*, 13(1), 21.	Wrong target group
28	Ould-Dada 2006. Dealing with uncertainty in the assessment of human exposure to radioactivity in food and the environment. *Environment International, 32*(8), 977-982.	No experimental results reported
29	Raimi et al. 2017. The Promise and Limitations of Using Analogies to Improve Decision-Relevant Understanding of Climate Change. *PLoS ONE* 12(1): e0171130.	Not focussing on questions relevant for us
30	Risbey et al. 2007. Expressions of likelihood and confidence in the IPCC uncertainty assessment process. *Climate Change*, 85, 19-31.	No experimental results reported
31	Rosenbaum et al. 2010. User testing and stakeholder feedback contributed to the development of understandable and useful Summary of Findings tables for Cochrane reviews. *Journal of Clinical Epidemiology, 63*(6), 607-619.	No experimental results reported

32	Santesso et al. 2016. Improving GRADE evidence tables part 3: detailed guidance for explanatory footnotes supports creating and understanding GRADE certainty in the evidence judgements. *Journal of Clinical Epidemiology, 74*, 28-39.	Not focussing on questions relevant for us
33	Shickle 2000. "On a supposed right to lie [to the public] from benevolent motives": Communicating health risks to the public. *Med Health Care Philos*, 3, 241.	Not focussing on questions relevant for us
34	Siebert et al. 2013. When is enough evidence enough? – Using systematic decision analysis and value-of-information analysis to determine the need for further evidence. *Zeitschrift für Evidenz, Fortbildung und Qualität im Gesundheitswesen, 107*(9), 575-584.	Not focussing on questions relevant for us
35	Spiegelhalter et al. 2011. Don't know, can't know: embracing deeper uncertainties when analysing risks. *Phil. Trans. R. Soc. A ,* 369, 4730–4750.	No experimental results reported
36	Spiegelhalter et al. 2011. Visualizing Uncertainty About the Future. *Science, 333*(6048), 1393-1400.	No experimental results reported
37	Spiegelhalter 2017. Risk and Uncertainty Communication. *Annu. Rev. Stat. Appl.*, 4, 31–60.	No experimental results reported
38	Stacey et al. 2014. Decision aids for people facing health treatment or screening decisions (Review). *Cochrane Database of Systematic Reviews*(4).	Not focussing on questions relevant for us
39	Steckelberg 2005. Dissertation. Retrieved from: http://ediss.sub.uni-hamburg.de/volltexte/2006/2744/pdf/Steckelberg.Dissertation.2005.final.pdf [29.04.2019].	Dissertation, not focussing on questions relevant for us
40	Van Dijk et al. 2007. Consumer responses to communication about food risk management. *Appetite, 50*(2), 340-352.	Methodological reasons (outcome variable makes no sense)
41	Vandvik, P. O., et al. (2012). Formatting modifications in GRADE evidence profiles improved guideline panelists comprehension and accessibility to information. A randomized trial. *Journal of clinical epidemiology*, 65(7), 748-755.	Wrong target group
42	Waltering et al. 2018. Kommunikation von Unsicherheit in Gesundheitsinformationen – eine geplante randomisierte kontrollierte Studie. EbM-Kongress, Graz 2018, DOI:10.13140/RG.2.2.16746.41926.	No experimental results reported
43	Wiedemann et al. 2013. Evidence maps – a tool for summarizing and communicating evidence in risk assessment. unpublished	No experimental results reported
44	Wiedemann et al. 2006. The Impacts of Precautionary Measures and the Disclosure of Scientific Uncertainty on EMF Risk Perception and Trust. *J Risk Res, 9*(4), 361-372.	Not focussing in question relevant for us
45	Woolf et al. 2011. Developing clinical practice guidelines: types of evidence and outcomes; values and economics, synthesis, grading, and presentation and deriving recommendations. *Implementation Science, 7*(1), 61.	No experiments reported
46	Zipkin et al. 2014. Evidence-Based Risk Communication: a systematic review. *Ann Intern Med, 161*(4), 270-280.	Not focussing on questions relevant for us

There is few empirical evidence available on the communication of quality of evidence/uncertainty. Three of the studies identified were literature reviews (Politi, Han, & Col, 2007; Schünemann et al., 2003; van der Bles et al., 2019)for which no quality assessment could be carried out (as of February 2018). More recently, the SANRA instrument (Baethge, Goldbeck-Wood, & Mertens, 2019) for assessing the quality of narrative reviews was published, but not applied retrospectively. In addition, four studies with a randomized design were identified (Akl et al., 2007; Bansback, Harrison, & Marra, 2016; Harrison, Marra, & Bansback, 2017; Santesso et al., 2016). The studies varied in their risk for bias (see Table 8 for quality ratings). Two of the studies (Akl et al., 2007; Bansback et al., 2016) are judged to be of concern in multiple domains that substantially lowers confidence in the results. The other two studies (Harrison et al., 2017; Santesso et al., 2016) are judged to be at low risk of bias in all domains. However, the study stimuli and outcomes were different, so that no overall risk of bias assessment was possible for the individual outcomes ("preference for a treatment" vs. "understanding of plain language information in general and of quality of evidence in specific" with regard to the results of systematic reviews).

Table 8: Summary of study quality ratings – Quality o evidence/ Uncertainty search

**Criteria**	**Akl et al. 2007**	**Harrison et al. 2017**	**Bansback et al. 2016**	**Santesso et al. 2015**
Random sequence generation (selection bias)	?	+	?	+
Allocation concealment (selection bias)	?	+	?	+
Blinding of participants and researchers (performance bias)	-	+	+	+
Blinding of outcome assessment (detection bias)	?	+	?	+
Incomplete outcome data (attrition bias)	+	+	+	+
Selective reporting (reporting bias)	+	+	+	+
Other bias	-	?	?	?
+ = Low risk of bias<br>- = High risk of bias<br>? = Unclear risk of bias				

Characteristics of the included studies are summarized in short in Table 9.

Summary of findings to inform risk profile development

In summary, although there are a number of recent literature reviews of how to communicate uncertainty, limited guidance exists on how to communicate quality of evidence:

- People are not averse to receiving uncertainty information, and it may actually improve their perceptions of the underlying evidence.
- There are very few studies testing different formats (e.g., numerical, symbolic, or visual displays of quality of evidence). Nevertheless, there are some suggestions from the literature on what symbol systems or categories could be tested.
- When non-quantifiable uncertainty is communicated, it can be communicated in a first case as a predefined categorization of uncertainty, otherwise as a qualifying verbal statement, or a list of possibilities or scenarios.

S1 File. Literature search (short report) – supplement material

Table 9: Summary of study characteristics – Quality of evidence/ Uncertainty search

Study, year	Objective	Study design	Search	Studies and sample	Intervention	Outcomes	Authors` conclusion
Akl et al. 2007	Assessments of health consumers' understanding, evaluations, and preferences for symbols compared to numbers and letters for the representation of strength of recommendations (SOR) and quality of evidence (QOE)	Questionnaire study in a randomized controlled design in the setting of a community health education program.	Not applicable	**Sample:** 84 participants from the general public with interest in health education (recruited at a session of the "Mini Medical School" at the State University of New York at Buffalo)	The session was a presentation on interpreting medical literature, containing explanations about the concepts of evidence, SOR, QOE, and health care recommendations. Participants were randomly assigned to one of two questionnaires (one focusing on symbols, the other one on numbers and letters), which tested participants' objective understanding of the presentation, and asked them to evaluate the presentation of SOR (ease of understanding, clearness and conciseness, and conveyance of uncertainty) and the presentation of QOE (ease of understanding, clearness and conciseness, and conveyance of QOE).	Objective understanding, evaluation, preference	Symbols were the better option for representing SOR. Letters were evaluated (slightly) more positively for conveying QOE than symbols, while objective understanding was high for both presentations. More studies are needed in order to draw conclusions about which forms of presentation are best, and to work toward a standardized system of grading QOE and recommendations. Guideline developers need to provide clear explanations for the forms of presentation used.

					At the end, they were shown both presentations and asked which one they preferred.		
Bansback et al. 2016	Evaluation of two methods for communicating imprecision in risks currently used in some patient decision aids and its influence on people's treatment decisions.	Randomized discrete choice experiment	Not applicable	**Sample:**<br>2.663 respondents from Canadian general population	Participants were randomized into 1 of 3 conditions with hypothetical treatment options for rheumatoid arthritis. Conditions differed in whether uncertainty information was provided: 1) no imprecision communicated, 2) imprecision communicated in the form of a confidence interval, including visual and 3) confidence in estimates described as "low", "moderate" or "high" ("very low" was omitted due to the fact that medical treatments with this level of quality of evidence would not be sold on the marked).	Understanding, magnitude of values, impact on decision uncertainty and conflict	Incorporating imprecision/uncertainty information in contexts where it differs between options can be important and could potentially influence treatment decisions. If imprecision is communicated, qualitative descriptions (e.g., describing confidence in results as low, moderate or high) should be preferred over the communication of confidence intervals, since this appears to be understood by more people. Both formats may slightly increase decision uncertainty and conflict compared to a format that leaves out any information on imprecision.
Harrison et al. 2017	Evaluation whether preferences for treatments that are labeled as "new" exist,	Randomized experiment (randomizing participants on two	Not applicable	Representative sample of 2837 Canadian adults from a market research panel, among	Three experiments that sought choices between hypothetical treatments for	Preference for the new treatment, depending on whether newness only, ambiguity only or	The communication of uncertainty/ ambiguity associated with the evidence base of a

	and if these preferences persist once ambiguity about the evidence base reflecting newness is described (new drugs tend to have a less certain evidence base).	levels: between formats and then between blocks within these formats)		other aspects, their innovativeness was assessed in a self-reported format with five categories (innovators, early adopters, early majority, late majority and laggards).	rheumatoid arthritis based on different levels of seven attributes (route and frequency of administration, chance of benefit, serious and minor adverse effects, life expectancy, and a "newness" attribute) were designed. The experiments differed in their description of the "newness" attribute: 1. description of treatment as new (recently available) or older (available for 5 or 10 years), 2. Use of a qualitative description of the confidence in the evidence about benefit and adverse-effects estimates, 3. Description of treatment as new or older plus a qualitative description of confidence in evidence.	newness and ambiguity were shown to participants.	medical treatment can diminish people's preference for treatments labelled as "new". In people who are already critical of such treatments labelled as "new", it can increase the preference for older treatments.
Politi et al. 2007	Review of the literature on various issues related to uncertainty in decision making: conceptualizing uncertainty, identifying its potential sources,	White paper	Not applicable	Not applicable	Not applicable	The literature was reviewed regarding cognitive and behavioral outcomes (information load, understanding, preferences, ambiguity aversion, information-	Research has not yet identified best practices for communicating uncertainty about harms and benefits of treatment. Practices depend on the task

	assessing uncertainty, potential methods of communicating uncertainty, potential outcomes of communicating uncertainty, and current practices and recommendations by expert groups on communicating uncertainty.					seeking behaviors (seeking or avoiding)); emotional outcomes: fear, anxiety, panic, desire to reduce uncertainty, heightened vigilance about illness, hope, optimism, regret, satisfaction; individual differences in patient responses to uncertainty: desired role in patient's medical care, values and preferences for medical care, personality traits, responses to physician's uncertainty.	required of the patient and the type of uncertainty. More conceptual, quantitative and qualitative research is needed to address this lack of knowledge.
Santesso et al. 2015	Evaluation of a new format to summarize results from Cochrane reviews for patients and the public in plain language.	Randomized controlled trial	Not applicable	143 members of the public from five countries (Canada, Norway, Spain, Argentina, and Italy)	Online intervention; Participants either received information about a health care intervention and its effects either in the old plain text summary format of Cochrane or in the new plain language format, which was not only more structured, but also used a question-answer format instead of plain text only and contained a table including information	Understanding, satisfaction with the summary, ease of understanding, and accessibility of the findings of the review	The new plain language format increased understanding of the information in general and of quality of evidence in specific. However, more research could be done to further improve the communication of uncertainty, since the number of participants who answered those questions correctly was still below 50%.

					on quality of evidence. Participants answered questions via an online questionnaire that assessed the outcomes; Formats were randomly allocated using block randomization.		
Schünemann et al. 2003	Evaluation on the communication of grades of evidence and strengths of recommendations in health care and discussion of advantages and disadvantages of using letters, numbers, symbols, and words to represent grades of evidence and recommendations.	Literature Review with essay	A search of MEDLINE and PsychLit databases for the period 1966 to April 2002 was conducted. In addition, authors searched for theoretical work and qualitative research addressing how best to communicate grades of any kind quickly and clearly. Furthermore, authors searched relevant texts and bibliographies and contacted researchers from other fields (e.g., psychology, marketing and graphic design). Because empirical evidence on the use of symbols comes from grading schemes unrelated to health care — such as Consumer Reports and restaurant and hotel guides — they also contacted	Not applicable, not empirical evidence identified.	Not applicable	Comprehension of different systems of communicating grades of evidence.	No relevant literature was found on the communication of grades of evidence in health care. If symbols are used, they should be universally and intuitively understood and be able to convey at least two dimensions. Further research needs to examine to what extent intended messages are comprehended before promoting a particular format.

			organizations responsible for popular grading schemes.				
van der Bles et al. 2019	Review on what is known about communicating epistemic uncertainty (uncertainty due to knowledge about current and past facts, numbers, or scientific models and hypotheses), and to propose a multidisciplinary framework that combines an analysis of uncertainty with one of communication, which could serve as a basis for future work.	Interdisciplinary review	Not applicable	Not applicable	Not applicable	Cognition, trust, affect, behavior.	An overarching framework that clarifies the components that make up the umbrella term uncertainty and those that comprise the process of communication, affecting an audience's reaction to uncertainty communication was developed:<br>• Who (people assessing the uncertainty, people doing the communication) communicates<br>• what (object, source, type, magnitude)<br>• in what form (expression of uncertainty, format, medium)<br>• to whom (characteristics of the audience, relationship audience to "what", relationship audience to "who")<br>• to what effect (cognition,

							emotion, trust, behavior).<br>More systematic research is necessary to identify effects of epistemic uncertainty communication on cognition, affect, trust, and behavior.

3.4. Search on 'How to communicate the dose-response relationship/thresholds'

The literature search on "How to communicate the dose-response relationship/thresholds" was conducted from 01-02-2018 to 14-02-2018. In addition to the search in four predefined databases (PubMed, PsycINFO, Web of Science, Scopus), we conducted a search in another database that could also include relevant studies to answer the question (ERIC via ProQuest). A search for gray literature was conducted in Google Scholar.

Search strategy – Dose-response search

**PubMed**			
**communication**	**dose-response**	**Substances/ Uncertainty**	**outcome**
(((communicat*[TIAB] OR "inform"[TIAB] OR "report"[TIAB] OR "educate"[TIAB]))) OR (("Communication"[Majr] OR "Consumer Health Information"[Majr] OR "Drug Labeling"[Majr]))	((("adverse effects" [Subheading] OR "No-Observed-Adverse-Effect Level"[Mesh]))) OR (("dose-response*"[TIAB] OR "dose effect"[TIAB] OR "benchmark"[TIAB] OR "dosage*"[TIAB] OR "limit value"[TIAB] OR "threshold"[TIAB]))	(((("Radiation"[Majr] OR "Environmental Pollution"[Majr] OR "Food and Beverages"[Majr] OR "Food Contamination"[Majr]))) OR (("food"[TIAB] OR "Consumer products"[TIAB] OR "chemicals"[TIAB] OR "chemical products"[TIAB]))) OR ((("toxic" OR harmful OR "danger*" OR "hazard*")) AND ("substance*" OR "product")) OR (uncertainty[TIAB] OR ambiguity[TIAB])	((("Knowledge"[Mesh] OR "Health Knowledge, Attitudes, Practice"[Majr] OR „Perception"[Mesh] OR "Comprehension"[Mesh] OR "Decision Making"[Mesh] OR "Patients"[Majr]))) OR ((Decision*[Title/Abstract] OR understanding[TIAB] OR "informed consent"[TIAB] OR layperson*[TIAB] OR Consumer*[TIAB] OR Experts[TIAB] OR students[TIAB] OR "decision maker"[TIAB] OR "stakeholder"[TIAB] OR "general public"[TIAB] OR Laypeople[TIAB] OR laymen[TIAB] OR "lay person"[TIAB] OR "non-experts"[TIAB] OR politicians[TIAB]))
180 results; MA SR, last 10 y: 133; RCTs, last 10y: 47			
**PsycINFO (Ovid)**			
**communication**	**dose-response**	**Substances /Uncertainty**	**outcome**
(communicat* OR inform* OR report* OR educat*).ti,ab. OR exp COMMUNICATION/	exp Drug Dosages/ or exp "Side Effects (Drug)"/ OR (No-Observed-Adverse-Effect Level OR dose-response* OR dose effect*	Exp RADIATION/ OR exp Environmental Effects/ OR exp FOOD/ OR exp FOOD SAFETY/ OR exp FOOD ADDITIVES/ OR exp	Exp health knowledge/ OR exp RISK PERCEPTION/ or exp PERCEPTION/ OR exp COMPREHENSION/ OR (decision*).ti,ab. OR exp

	OR benchmark Adj2 dos* OR dosage* OR limit value* OR threshold*).ti,ab.	CHEMICALS/ OR (Consumer products).ti,ab. OR ((toxic OR harmful OR danger OR hazard) AND (substance* OR product*)).ti,ab. OR (uncertainty OR ambiguity).ti,ab.	Behavior/ OR (informed adj2 (decision* or choice*)).ti,ab. OR (layperson* OR Consumer* OR Experts OR students OR decision maker OR stakeholder OR general public OR Laypeople OR laymen OR lay person OR non-experts OR politicians).ti,ab.
36 results; MA SR, last 10 y: 3; RCTs, last 10y: 33 (randomised controlled trial*.mp. OR randomized controlled trial.mp. OR random sampling/ OR experiment controls/ OR exp Treatment Effectiveness Evaluation/ OR controlled stud*.mp. OR controlled experiment*.mp. OR random*.mp. OR exp Clinical Trials/)			
**Web of Science**			
**communication**	**dose-response**	**Substances/ Uncertainty**	**outcome**
TS=((communication) OR (“health information”) OR (communicat* OR inform* OR report* OR educat*))	TS=(“Drug dosage*” OR “side effect*” OR “adverse effect*” OR “dose effect*” OR “dose response*” OR “benchmark” OR “dosage*” OR “limit value*” OR “threshold*” OR “no observed effect level”)	TS=(radiation OR “Environmental Effect*” OR Food OR “Food Safety” OR Chemicals OR “Consumer product*” OR ((toxic OR harmful OR danger* OR hazard*) NEAR (substance* OR product*)) OR uncertainty OR ambiguity)	TS=(“health knowledge” OR Perception OR Comprehension OR decision* OR Behavior OR (informed NEAR (decision* or choice*)) OR layperson* OR Consumer* OR Experts OR students OR decision maker OR stakeholder OR “general public” OR Laypeople OR laymen OR “lay person” OR “non-experts” OR politicians)
539 results; MA SR, last 10 y: 246 (TS=(„Systematic Review“ OR Metaanalysis OR „Meta-analysis“); RCTs, last 10y: 293 (TS=(“41andomized controlled trial*” OR “randomized controlled trial*” OR (experiment NEAR (control* OR random*)) OR “controlled stud*”))			
**Scopus**			
**communication**	**dose-response**	**Substances /Uncertainty**	**outcome**
TITLE-ABS (communicat* OR “health information” OR communicat* OR inform* OR report* OR educat*)	TITLE-ABS (“Drug dosage*” OR “side effect*” OR “adverse effect*” OR “dose effect*” OR “dose response*” OR “benchmark” OR “dosage*”	TITLE-ABS (radiation OR “Environmental Effect*” OR Food OR “Food Safety” OR Chemicals OR “Consumer product*” OR ((toxic OR harmful OR danger* OR	TITLE-ABS (“health knowledge” OR Perception OR Comprehension OR decision* OR Behavior OR (informed AND (decision*

	OR "limit value*" OR "threshold*" OR "no observed effect level")	hazard*) AND (substance* OR product*)) OR uncertainty OR ambiguity)	or choice*)) OR layperson* OR Consumer* OR Experts OR students OR "decision maker" OR stakeholder OR "general public" OR Laypeople OR laymen OR "lay person" OR "non-experts" OR politicians)
56 results; MA SR, last 10 y: 15: (TITLE-ABS-KEY ("Systematic Review" OR Metaanalysis OR "Meta-analysis"); RCTs, last 10y: 41 (TITLE-ABS-KEY ("42andomized controlled trial*" OR "randomized controlled trial*" OR "random sampling" OR (experiment AND (control OR study)) OR "Effectiveness Evaluation" OR "controlled stud*" OR "controlled experiment*" OR random* OR "Clinical Trial")			
**Eric**			
**communication**	**dose-response**	**Substances /Uncertainty**	**outcome**
MAINSUBJECT.EXACT("Non verbal Communication") OR MAINSUBJECT.EXACT("Communication (Thought Transfer)") OR MAINSUBJECT.EXACT("Audiovisual Communications") OR MAINSUBJECT.EXACT("Communication Strategies") OR ab,ti(communicat* OR "health information" OR inform* OR report* OR educat*)	ab,ti("Drug dosage*" OR "side effect*" OR "adverse effect*" OR "dose effect*" OR "dose response*" OR "benchmark" OR "dosage*" OR "limit value*" OR "threshold*" OR "no observed effect level")	MAINSUBJECT.EXACT("Hazardous Materials") OR ab,ti(radiation OR "Environmental Effect*" OR Food OR "Food Safety" OR Chemicals OR "Consumer product*" OR uncertainty OR ambiguity) OR ab,ti((toxic OR harmful OR danger* OR hazard*) AND (substance* OR product*))	MAINSUBJECT.EXACT("Learning") OR MAINSUBJECT.EXACT("Comprehension") OR MAINSUBJECT.EXACT("Decision Making") OR MAINSUBJECT.EXACT("Participative Decision Making") OR MAINSUBJECT.EXACT("Behavior") OR ab,ti(knowledge OR Perception OR (informed AND (decision* or choice*)) OR layperson* OR Consumer* OR Experts OR students OR "decision maker" OR stakeholder OR "general public" OR Laypeople OR laymen OR "lay person" OR "non-experts" OR politicians)
Results: 5; MA SR, last 10 y: 0 (ab,ti("Systematic Review" OR Metaanalysis OR "Meta-analysis"); RCTs, last 10y: 5 (ab,ti("42andomized controlled trial*" OR "randomized controlled trial*" OR "random sampling" OR (experiment AND (control OR study)) OR "Effectiveness Evaluation" OR "controlled stud*" OR "controlled experiment*" OR random* OR "Clinical Trial")			

S1 File. Literature search (short report) – supplement material

Changes in inclusion/exclusion criteria

We included studies on the communication of dose-response relationships/thresholds in food, consumer, or chemical products. Of relevance were primary or secondary publications reporting on experiments that evaluated different strategies for communicating dose-response relationships/thresholds in different target groups (e.g., general public, experts, stakeholder). Because dose-response relationships are influenced by various factors and there is uncertainty associated with the toxicity of various substances, we also searched for ways to communicate uncertainty in these relationships.

Results

The literature search revealed a total of 816 hits: 180 from PubMed, 36 from PsycINFO (Ovid), 539 from Web of Science and 56 from Scopus and five from ERIC. After removal of duplicates, 777 publications were left. After title and abstract screening, no publication was left for full-text screening. The gray literature search revealed no publication on this question (see **Figure 4**).

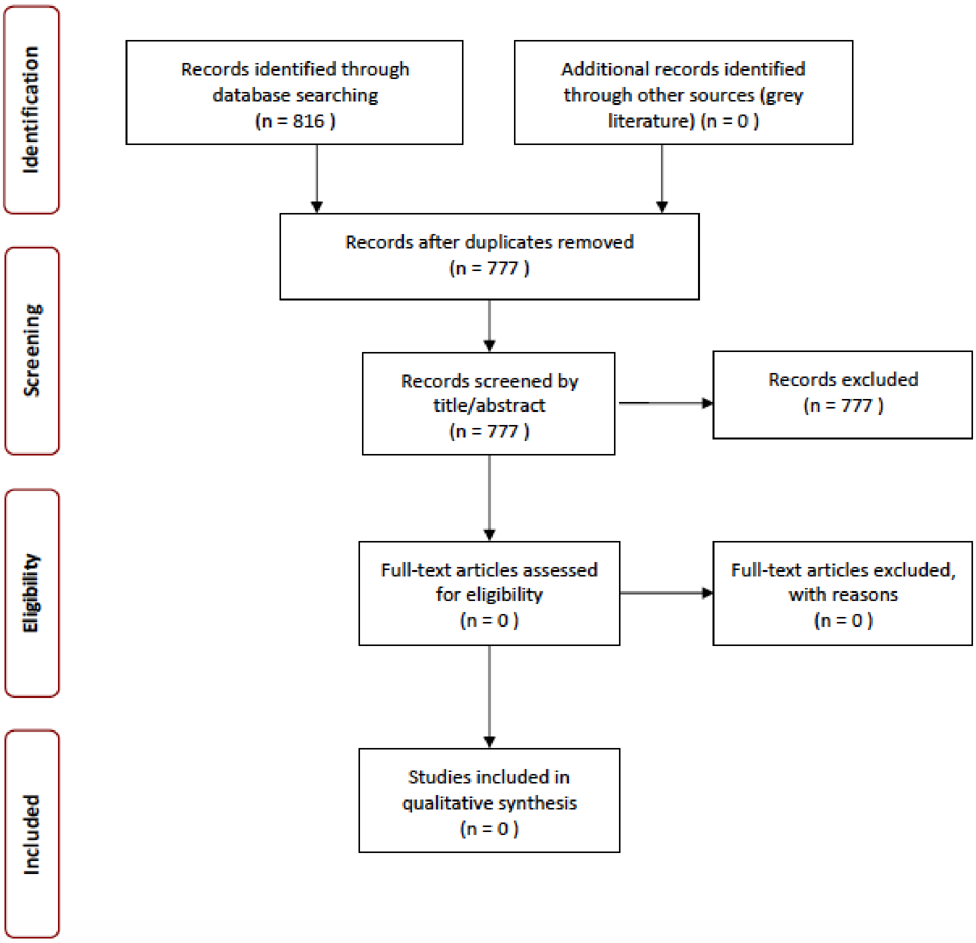

Figure 4: Study selection flow chart - Dose-response search

Studies that were identified focused mainly on risk assessment results for specific substances or products and on how thresholds or limit values were derived. No publications on the general communication of dose-response relationships or thresholds were identified.

3.5. Search on 'How to communicate the severity of health impairments'

The literature search on "How to communicate the severity of health impairments" was conducted from 20-02-2018 to 22-02-2018. We searched in four predefined databases (PubMed, PsycINFO, Web of Science, Scopus).

Search strategy – Severity search

**PubMed**		
**communication**	**severity**	**outcome**
((visual*[TIAB] OR verbal*[TIAB] OR numeric*[TIAB] OR graphic*[TIAB]) AND (communicat*[TIAB] OR display*[TIAB] OR information [TIAB] OR represent*[TIAB] OR presentat*[TIAB])) OR "Communication"[Mesh] OR "Health Communication"[Mesh] OR "Nonverbal Communication"[Mesh] OR "Consumer Health Information"[Majr] OR "health information" [TIAB] OR "Decision Support Techniques"[Mesh] OR ((decision*[TIAB]) AND (tool [TIAB] OR material*[TIAB]))	"Drug-Related Side Effects and Adverse Reactions"[Majr] OR (adverse [TIAB] OR severe [TIAB] OR irreversible [TIAB] OR reversible [TIAB]) AND<br><br>"health impairment*"[TIAB] OR "health impact*"[TIAB] OR "health consequence*"[TIAB] OR "health damage*"[TIAB] OR "health effect*"[TIAB] OR "health outcome*"[TIAB] OR "side effect*"[TIAB] OR "severity of illness" [TIAB] OR "severity of harm*"[TIAB] OR "disease severity" [TIAB]	"Knowledge"[Mesh] OR "Health Knowledge, Attitudes, Practice"[Majr] OR "Perception"[Mesh] OR "Comprehension"[Mesh] OR "Decision Making"[Mesh] OR attitude [TIAB] OR awareness [TIAB] OR perception [TIAB] OR knowledge [TIAB] OR "shared-decision"[TIAB] OR behaviour [TIAB] OR behavior [TIAB] OR "informed decision"[TIAB] OR "informed decisions" [TIAB] OR "informed choice" [TIAB] OR "informed choices" [TIAB] OR "decision-making"[TIAB]
MA SR, last 10 y: 58; RCTs, last 10y: 53		
**PsycINFO**		
**communication**	**severity**	**outcome**
exp VERBAL COMMUNICATION/ or exp Graphical Displays/ or exp NONVERBAL COMMUNICATION/ or exp WRITTEN COMMUNICATION/ or exp COMMUNICATION/ or exp ORAL COMMUNICATION/ or ((visual* or verbal* or numeric* or graphic*) and (communicat* or display* or information or represent* or presentat*)).ti,ab OR health information.ti,ab. OR (decision* and (tool or material*)).ti,ab	exp "Side Effects (Drug)"/ OR exp Health Impairments/ OR ((adverse OR severe OR irreversible OR reversible) AND<br><br>(health impairment* OR health impact* OR health consequence* OR health damage* OR health effect* OR health outcome* OR side effect*)) OR severity of illness.ti,ab. OR severity of harm*.ti,ab OR disease severity.ti,ab.	exp health knowledge/ OR<br><br>exp RISK PERCEPTION/ or exp PERCEPTION/ OR<br><br>exp COMPREHENSION/ OR<br><br>risk literacy.ti,ab. OR<br><br>attitude.ti,ab. OR<br><br>exp AWARENESS/ OR<br><br>Patient Participation.ti,ab. OR (shared adj2 decision).ti,ab. OR<br><br>(decision* adj2 (conflict* OR regret*)).ti,ab. OR<br><br>exp Behavior/ OR<br><br>(informed adj2 (decision* or choice*)).ti,ab.

S1 File. Literature search (short report) – supplement material

<table>
<tr><td colspan="3">MA SR: 7; RCTs, last 10y: 35 (randomised controlled trial*.mp. OR randomized controlled trial.mp. OR random sampling/ OR experiment controls/ OR exp Treatment Effectiveness Evaluation/ OR controlled stud*.mp. OR controlled experiment*.mp. OR random*.mp. OR exp Clinical Trials/)</td></tr>
<tr><td colspan="3">Web of Science</td></tr>
<tr><td>communication</td><td>severity</td><td>outcome</td></tr>
<tr><td>#1 TS=(communication) OR

#2 TS=((visual* or verbal* or numeric* or graphic*) NEAR (communicat* or display* or information or represent* or presentat*)) OR

#3 Health Communication OR

#4 Health Information OR

#5 ((decision*) NEAR (tool OR material*))

#6 #1 OR #2 OR #3 OR #4 OR #5</td><td>#7 TS= ((adverse OR severe OR irreversible OR reversible) NEAR ("health impairment*" OR "health impact*" OR "health consequence*" OR "health damage*" OR "health effect*" OR "health outcome*" OR "side effect*"))

#8 “severity of illness” OR

#9 “severity of harm*” OR

#10 “disease severity”

#11 #7 OR #8 OR #9 OR #10</td><td>#12 TS=(knowledge OR perception OR comprehension OR "risk literacy" OR behavior OR "decision making" OR awareness OR "shared decision" OR "informed decision" OR "informed choice")

#13 #6 AND #11 AND #12</td></tr>
<tr><td colspan="3">Indexes=SCI-EXPANDED, SSCI, A&HCI, CPCI-S, CPCI-SSH, BKCI-S, BKCI-SSH, ESCI, CCR-EXPANDED, IC Timespan=2012-2017</td></tr>
<tr><td colspan="3">MA SR, last 10 y: 24; RCTs, last 10y: 51 (TS=("randomised controlled trial*" OR "randomized controlled trial*" OR "random sampling" OR (experiment NEAR (control OR study)) OR "Effectiveness Evaluation" OR "controlled stud*" OR "controlled experiment*" OR random* OR "Clinical Trial"))</td></tr>
<tr><td colspan="3">Scopus</td></tr>
<tr><td>communication</td><td>severity</td><td>outcome</td></tr>
<tr><td>#1 TITLE-ABS-KEY ((visual* OR verbal* OR numeric* OR graphic*) PRE/5 (communicat* OR display* OR information OR represent* OR presentat*))

#2 TITLE-ABS-KEY ((decision*) PRE/5 (tool OR material*))</td><td># 5 TITLE-ABS-KEY ((adverse OR severe OR irreversible OR reversible) PRE/5 ("health impairment*" OR "health impact*" OR "health consequence*" OR "health damage*" OR "health effect*" OR "health outcome*" OR "side effect*"))</td><td>#8 TITLE-ABS-KEY (knowledge OR perception OR comprehension OR "risk literacy" OR behavior OR "decision making" OR awareness OR "shared decision" OR "informed decision" OR "informed choice")

#9 #4 AND #7 AND #8</td></tr>
</table>

#3 TITLE-ABS-KEY ("health information")<br><br>#4 #1 OR #2 OR #3	#6 TITLE-ABS-KEY ("severity of illness" OR "severity of harm*" OR "disease severity")<br><br>#7 #5 OR #6	#10 #9 AND NOT INDEX (medline) AND PUBYEAR > 2007
MA SR, last 10 y: 64 (ALL ("Systematic Review" OR "Metaanalysis" OR "Meta-analysis")); RCTs, last 10y: 79 (ALL("randomised controlled trial*" OR "randomized controlled trial*" OR "random sampling" OR (experiment AND (control OR study)) OR "Effectiveness Evaluation" OR "controlled stud*" OR "controlled experiment*" OR random* OR "Clinical Trial"))		

Changes in inclusion/exclusion criteria

We have not made any changes to the predefined inclusion/exclusion criteria.

Results

The literature search revealed a total of 342 hits: 82 from PubMed, 42 from PsycINFO (Ovid), 75 from Web of Science, and 143 from Scopus. Two studies were identified by gray literature search. After removal of duplicates, 334 publications were left. After title and abstract screening, 11 publications were left for full-text screening. No systematic reviews or meta-analyses on how to communicate the severity of health impairments were identified. Therefore, 11 primary studies were included for full text-screening. After exclusion of eight publications with reason (see **Table 10**), three studies were included for data synthesis (see **Figure 5**).

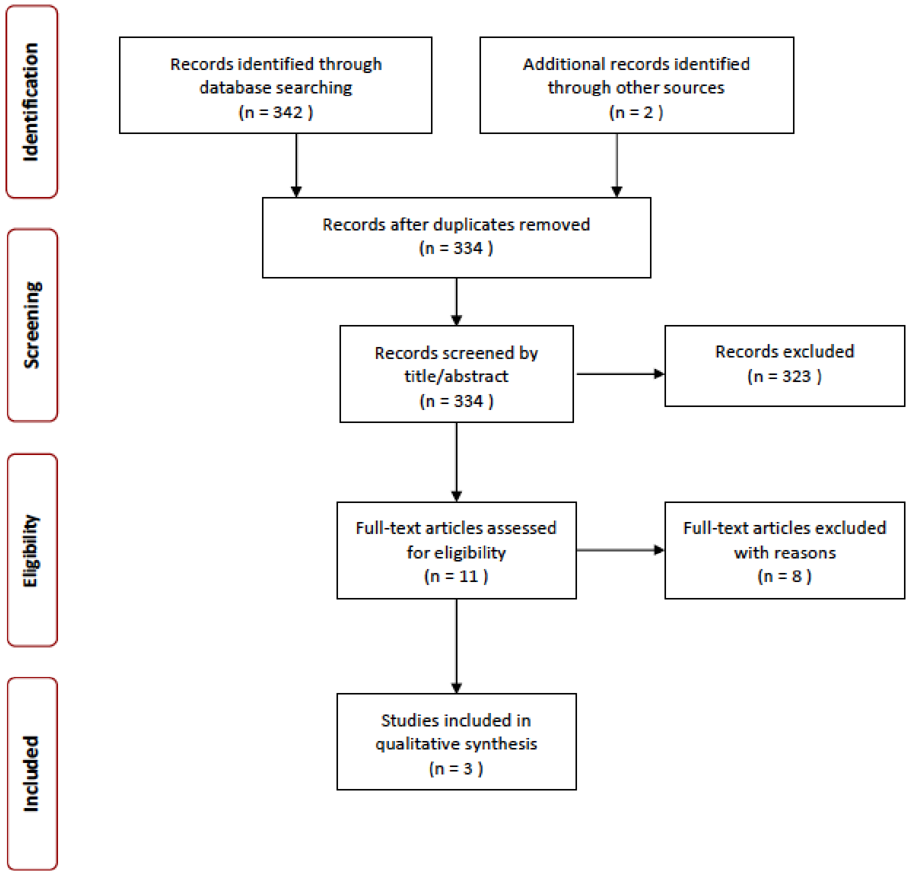

Figure 5: Study selection flow chart - Severity search

Table 10: Studies excluded with reason – Severity search

	Study	Exclusion reason
1	Ancker, J. S., et al. (2011). "Effects of Game-Like Interactive Graphics on Risk Perceptions and Decisions." *Med Decis Making* 31(1): 130-142.	Wrong focus: visualisation of risks was tested, no information about severity was tested
2	Eyler, R. F., et al. "Utilization of Continuous "Spinners" to Communicate Risk." *Med Decis Making* 2017 Aug;37(6):725-729.	Wrong focus: no information about severity was tested
3	Knapp, P., et al. (2009). "Perceived risk of medicine side effects in users of a patient information website: A study of the use of verbal descriptors, percentages and natural frequencies." *Br J Health Psychol* 14(3): 579-594.	Wrong focus: probability presentation was tested, no information about severity was tested
4	Knapp, P., et al. "Perceived risk of tamoxifen side effects: a study of the use of absolute frequencies or frequency bands, with or without verbal descriptors." *Patient Educ Couns* 2010 May;79(2):267-71.	Wrong focus: numerical vs. verbal (and combined) frequency information was tested, no information about severity was tested
5	Knapp, P., et al. (2016). "Combined verbal and numerical expressions increase perceived risk of medicine side-effects: A randomized controlled trial of ema recommendations." *Health Expect* 19(2): 264-274.	Wrong focus: numerical vs. verbal (and combined) frequency information was tested, no information about severity was tested
6	Peters, E., et al. "Informing patients: the influence of numeracy, framing, and format of side effect information on risk perceptions." *Med Decis Making* 2011 May-Jun;31(3):432-6.	Wrong focus: numerical vs. verbal (and combined) frequency information and framing was tested, no information about severity was tested
7	Weinstein, ND. (200). "Perceived probability, perceived severity, and health-protective behavior." *Health Psychol*. Vol. 19, No. 1, 65-74.	Case study
8	Zikmund-Fisher, B. J., et al. (2008). "Communicating side effect risks in a tamoxifen prophylaxis decision aid: The debiasing influence of pictographs." *Patient Educ Couns* 73(2): 209-214.	Wrong focus: numerical, verbal, visual presentation of side effect information was tested, no information about severity was tested

The methodological quality of the included studies was moderate (see **Table 11**). For example, sample size justification, power description, or variance and effect estimates were not provided in two studies (R. Sawant & Sansgiry, 2018; Seyed-Hosseini et al., 2010). Exposure was not assessed more than once over time in all three studies. In one study outcome assessors were not blinded to the exposure status of participants and key potential confounding variables were not measured and adjusted statistically for their impact on the relationship between exposure and outcome (Seyed-Hosseini et al., 2010).

Table 11: Summary of study quality ratings – Severity search

**Criteria**	**Sawant & Sansgiry 2018**	**Sawant et al. 2016**	**Seyed-Hosseini et al. 2010**
1. Was the research question or objective in this paper clearly stated?	Y	Y	Y
2. Was the study population clearly specified and defined?	Y	Y	Y
3. Was the participation rate of eligible persons at least 50%?	Y	Y	NA
4. Were all the subjects selected or recruited from the same or similar populations (including the same time period)? Were inclusion and exclusion criteria for being in the study prespecified and applied uniformly to all participants?	Y	Y	Y
5. Was a sample size justification, power description, or variance and effect estimates provided?	N	Y	N
6. For the analyses in this paper, were the exposure(s) of interest measured prior to the outcome(s) being measured?	Y	Y	NA
7. Was the timeframe sufficient so that one could reasonably expect to see an association between exposure and outcome if it existed?	Y	NA	Y
8. For exposures that can vary in amount or level, did the study examine different levels of the exposure as related to the outcome (e.g., categories of exposure, or exposure measured as continuous variable)?	NA	Y	N
9. Were the exposure measures (independent variables) clearly defined, valid, reliable, and implemented consistently across all study participants?	Y	Y	Y
10. Was the exposure(s) assessed more than once over time?	N	N	N
11. Were the outcome measures (dependent variables) clearly defined, valid, reliable, and implemented consistently across all study participants?	Y	Y	Y
12. Were the outcome assessors blinded to the exposure status of participants?	NR	NA	N
13. Was loss to follow-up after baseline 20% or less?	NA	NA	NA
14. Were key potential confounding variables measured and adjusted statistically for their impact on the relationship between exposure(s) and outcome(s)?	Y	Y	N
*Y=Yes; N=No; CD=cannot determine; NR=not reported; NA=not applicable			

Characteristics of the included studies are summarized in short in **Table *12***.

Summary of findings to inform risk profile development

In summary, there are only a few studies on the question of how to communicate severity of health impairments, and we can only derive a few recommendations:

- Because risk perception depends on both the severity and the probability of health impairment, it should be communicated for each concrete impairment that can occur.
- To validate the severity of the side effects (low, moderate, or high), the target group should be included.
- In order to avoid different perceptions of verbal descriptors of risk likelihood, exact numbers should be provided.

S1 File. Literature search (short report) – supplement material

Table 12 Summary of study characteristics - Severity search

Study, year	Objective	Study design	Sample	Intervention	Outcomes	Authors` conclusion
Sawant et al. 2016	Evaluation of different side effect communication styles on risk perception	Experimental cross-sectional factorial design	100 Pharmacy students from USA	Different drug information styles were tested (frequency verbal or numbers alone. Each participant received information about four medication side effects (one for each drug), where the descriptions were randomized to either verbal or the combined communication style with either a high or low frequency and a mild or severe associated severity with the side effect.	Risk perception	Providing information in a combined communication style helped to differentiate between low and high frequency side effects. Communication style aided in understanding of the underlying context of the side effect.
Sawant & Sansgiry 2018	Evaluation of different side effect communication styles on risk perception of side-effects in the light of contextual factors of rate and severity	Experimental cross-sectional factorial design	196 participants from the general public (mean age 42 years; 53% females, 58% non-Hispanic White, 18% African Americans, 16% Hispanic, 7% Asian, 57% college education, 32% masters or doctoral degree, 81% non-healthcare profession	Different drug information styles were tested. The factorial design consisted of three factors with two levels each (2x2x2 factorial). The three factors were communication format, side-effect rate and side-effect severity. The information about side-effects was presented to the participants as a component of a drug information box (DIB) with deidentified labels. The eight combinations were divided into two groups.	Risk perception, perception of severity	The effect of communication format on risk perception was significantly impacted by the underlying rate of occurrence. Risk of low rate side effects may be over-estimated when words-only descriptions are used and hence should be carefully communicated. Overall, use of words + numeric descriptors lead to better understanding of the risk and should be routinely incorporated in communication resources.

				Each participant was randomly assigned to one of the two groups. Thus, each participant received four DIBs based on the group assignment, and each DIB contained information on one side-effect presented using a combination of the three factors.		
Seyed-Hosseini et al. 2010	Evaluation on how the provision of numerical information on side effects influences the decision of OTC drug users.	Mixed-methods-Design (experimental factorial design with two repetitions, semi-structured interviews)	30 participants from Saskatoon (Canada) aged 51 to 89 years (average 66,6 years), 19 females. Most participants had a high school diploma or higher, 5 had fewer school years. 23 currently were using OTC, and 23 were taking prescription drugs. All participants had some physical or emotional health problems. REALM data showed that all participants were able to read most patient education materials.	Information about side effects and efficiency of three drugs against headache were presented to the participants twice, first without information about the frequency of side effects, then with numerical information about the frequency of side effects. 50% of participants received a scenario in which the headache was described as rare but severe. The others were given a scenario with a mild headache which was common. Participants had to choose one (or no) drug. Before and after the study, participants were asked whether they would prefer the presentation of side effects only, percentages of side	Risk perception, decision making depending on numerical information on risk	The provision of numerical information on the frequency of side effects lead to relief in the participants. Before, the risk was overestimated. After knowing the percentages of side effects the effectiveness of the drugs became more important in the process of decision making.

				effects, or whether they had no preference.		

4. References

Akl, E. A., Maroun, N., Guyatt, G., Oxman, A. D., Alonso-Coello, P., Vist, G. E., . . . Schünemann, H. J. (2007). Symbols were superior to numbers for presenting strength of recommendations to health care consumers: a randomized trial. *Journal of clinical epidemiology, 60*(12), 1298-1305.

Baethge, C., Goldbeck-Wood, S., & Mertens, S. (2019). SANRA—a scale for the quality assessment of narrative review articles. *Research integrity and peer review, 4*(1), 1-7.

Bansback, N., Harrison, M., & Marra, C. (2016). Does introducing imprecision around probabilities for benefit and harm influence the way people value treatments? *Medical Decision Making, 36*(4), 490-502.

Barros, I. M. C., Alcântara, T. S., Mesquita, A. R., Santos, A. C. O., Paixão, F. P., & Lyra, D. P., Jr. (2014). The use of pictograms in the health care: A literature review. *Research in Social & Administrative Pharmacy, 10*(5), 704-719. doi: 10.1016/j.sapharm.2013.11.002

Blümle, A., Lagrèze, W., & Motschall, E. (2018). Systematische Literaturrecherche in PubMed. *Der Ophthalmologe, 115*(3), 243-260.

Büchter, R. B., Fechtelpeter, D., Knelangen, M., Ehrlich, M., & Waltering, A. (2014). Words or numbers? Communicating risk of adverse effects in written consumer health information: A systematic review and meta-analysis. *BMC Medical Informatics and Decision Making, 14*(1). doi: 10.1186/1472-6947-14-76

EFSA (European Food Safety Authority), Hart, A., Maxim, L., Von Goetz, N., da Cruz, C., Merten, C., . . . Hardy, A. (2019). Guidance on Communication of Uncertainty in Scientific Assessments. *EFSA Journal, 17*(1), 5520. doi: 10.2903/j.efsa.2019.5520

Fitzpatrick-Lewis, D., Yost, J., Ciliska, D., & Krishnaratne, S. (2010). Communication about environmental health risks: a systematic review. *Environ Health, 9*, 67. doi: 10.1186/1476-069x-9-67

French, D. P., Cameron, E., Benton, J. S., Deaton, C., & Harvie, M. (2017). Can communicating personalised disease risk promote healthy behaviour change? A systematic review of systematic reviews. *Annals of Behavioral Medicine*. doi: 10.1007/s12160-017-9895-z

Frewer, L. J., Fischer, A. R., Brennan, M., Banati, D., Lion, R., Meertens, R. M., . . . Vereijken, C. M. (2016). Risk/Benefit Communication about Food-A Systematic Review of the Literature. *Crit Rev Food Sci Nutr, 56*(10), 1728-1745. doi: 10.1080/10408398.2013.801337

Garcia-Retamero, R., & Cokely, E. T. (2017). Designing visual aids that promote risk literacy: A systematic review of health research and evidence-based design heuristics. *Human Factors, 59*(4), 582-627. doi: 10.1177/0018720817690634

Gates, A., Gates, M., Duarte, G., Cary, M., Becker, M., Prediger, B., . . . Hartling, L. (2018). Evaluation of the reliability, usability, and applicability of AMSTAR, AMSTAR 2, and ROBIS: protocol for a descriptive analytic study. *Systematic reviews, 7*(1), 85.

Hallgreen, C. E., Mt-Isa, S., Lieftucht, A., Phillips, L. D., Hughes, D., Talbot, S., . . . Hermann, R. (2016). Literature review of visual representation of the results of benefit–risk assessments of medicinal products. *Pharmacoepidemiol Drug Saf, 25*(3), 238-250.

Harrison, M., Marra, C. A., & Bansback, N. (2017). Preferences for 'new'Treatments Diminish in the Face of Ambiguity. *Health economics, 26*(6), 743-752.

Harrison, M., Rigby, D., Vass, C., Flynn, T., Louviere, J., & Payne, K. (2014). Risk as an attribute in discrete choice experiments: A systematic review of the literature. *The Patient: Patient-Centered Outcomes Research, 7*(2), 151-170. doi: 10.1007/s40271-014-0048-1

Higgins, J. P., Altman, D. G., Gøtzsche, P. C., Jüni, P., Moher, D., Oxman, A. D., . . . Sterne, J. A. (2011). The Cochrane Collaboration's tool for assessing risk of bias in randomised trials. *Bmj, 343*, d5928.

Lühnen, J., Albrecht, M., Mühlhauser, I., & Steckelberg, A. (2017). Leitlinie evidenzbasierte Gesundheitsinformation. from www.leitlinie-gesundheitsinformation.de

Moher, D., Liberati, A., Tetzlaff, J., Altman, D. G., & Group, P. (2009). Preferred reporting items for systematic reviews and meta-analyses: the PRISMA statement. *PLoS medicine, 6*(7), e1000097. doi: 10.1371/journal.pmed.1000097

Mt-Isa, S., Hallgreen, C. E., Asiimwe, A., Downey, G., Genov, G., Hermann, R., & Tzoulaki, I. (2013). Review of visualisation methods for the representation of benefit-risk assessment of medication: Stage 2 of 2. *London, UK: PROTECT Consortium*.

National Heart, L., & Institute, B. (2014). Quality assessment tool for observational cohort and cross-sectional studies. *Bethesda: National Institutes of Health, Department of Health and Human Services*.

Politi, M. C., Han, P. K., & Col, N. F. (2007). Communicating the uncertainty of harms and benefits of medical interventions. *Medical Decision Making, 27*(5), 681-695.

Santesso, N., Carrasco-Labra, A., Langendam, M., Brignardello-Petersen, R., Mustafa, R. A., Heus, P., . . . Sinclair, D. (2016). Improving GRADE evidence tables part 3: detailed guidance for explanatory footnotes supports creating and understanding GRADE certainty in the evidence judgments. *Journal of clinical epidemiology, 74*, 28-39.

Sawant, R., & Sansgiry, S. (2018). Communicating risk of medication side-effects: role of communication format on risk perception. *Pharm Pract (Granada), 16*(2), 1174. doi: 10.18549/PharmPract.2018.02.1174

Sawant, R. V., Beatty, C. R., & Sansgiry, S. S. (2016). Effect of Communication Style on Perceptions of Medication Side Effect Risk among Pharmacy Students. *American Journal of Pharmaceutical Education, 80*(8), 131.

Schünemann, H. J., Best, D., Vist, G., Oxman, A. D., & Group, G. W. (2003). Letters, numbers, symbols and words: how to communicate grades of evidence and recommendations. *Cmaj, 169*(7), 677-680.

Seyed-Hosseini, M., Taylor, J., & Quest, D. (2010). - Discussing side effects of over-the-counter medicines: Impact of adding percentage data. - *18*(- 5), - 281.

Shea, B. J., Grimshaw, J. M., Wells, G. A., Boers, M., Andersson, N., Hamel, C., . . . Bouter, L. M. (2007). Development of AMSTAR: a measurement tool to assess the methodological quality of systematic reviews. *BMC medical research methodology, 7*(1), 10.

Stellamanns, J., Ruetters, D., Dahal, K., Schillmoeller, Z., & Huebner, J. (2017). Visualizing risks in cancer communication: A systematic review of computer-supported visual aids. *Patient Education and Counseling*. doi: 10.1016/j.pec.2017.02.003

van der Bles, A. M., van der Linden, S., Freeman, A. L., Mitchell, J., Galvao, A. B., Zaval, L., & Spiegelhalter, D. J. (2019). Communicating uncertainty about facts, numbers and science. *Royal Society open science, 6*(5), 181870.

West, S. L., Squiers, L. B., McCormack, L., Southwell, B. G., Brouwer, E. S., Ashok, M., . . . Sullivan, H. W. (2013). Communicating quantitative risks and benefits in promotional prescription drug labeling or print advertising. *Pharmacoepidemiol Drug Saf, 22*(5), 447-458. doi: 10.1002/pds.3416

Zipkin, D. A., Umscheid, C. A., Keating, N. L., Allen, E., Aung, K., Beyth, R., . . . Feldstein, D. A. (2014). Evidence-Based Risk Communication A Systematic Review. *Annals of Internal Medicine, 161*(4), 270-+. doi: 10.7326/M14-0295
