# Supplementary material for: Identifying content to improve risk assessment communications within the Risk Profile: Literature reviews and focus groups with expert and non-expert stakeholders
Source: PLoS One. 2022 Apr 11;17(4):e0266800. doi: 10.1371/journal.pone.0266800 (PMC9000125; doi:10.1371/journal.pone.0266800)
Supplement: S3 File — (PDF) [file pone.0266800.s003.pdf]

S3 File. Focus group interviews – supplement material

Ellermann C, McDowell M, Schirren C, Lindemann AK, Koch S, Lohmann M, Jenny MA. Identifying content to improve risk assessment communications within a Risk Profile: Literature reviews and Focus groups with expert and non-expert stakeholders

1. Methods

A question guide for each target group was developed by the HC a priori in consultation with the BfR. In order to give the interviewees as much freedom as possible and to allow for the free flow of speech, semi-structured questionnaires with open questions were used. The interviewer was reserved and only became active if no participant had an addition to a question or if the conversation stopped prematurely.

Ethics

Ethics approval for each interview was obtained from the Max Planck Institute for Human Development (Max-Planck-Institut für Bildungsforschung – MPIB). Written declaration of consent to participate in the study and for audio recording was obtained from all participants before the focus groups' interviews commenced.

Participant statements were anonymised during transcription. Personal contact data and transcription data are stored separately and treated as strictly confidential.

Data Collection

Five focus group were conducted with authors and diverse end-users of risk assessment. Semi-structured interview guides were developed for each target group. The interviews were audio-recorded and field notes were taken.

All focus group interviews were conducted by two moderators (CE, CS) who were involved in the study from the beginning on and accompanied by scientists from the BfR (AKL, ML). Both moderators had no previous experience in moderating and analysing focus group interviews, but were fully briefed by the team's supervisors.

Participants' characteristics, such as gender, age, country of origin, and degree, were asked only in the lay interviews to identify any differences in needs, preferences, and understanding between a higher-educated group and a lower-educated group, and were asked at the end of the questionnaire.

Recruitment

Participants of the focus groups with risk assessors and risk managers were contacted directly by the BfR. Participants for the lay population group interviews were recruited by an external market research company commissioned by the BfR.

Transcription

The interviews were documented with the help of audio recording devices to facilitate transcription. The software *f4transkript* was used for the transcription of the focus groups' interviews with risk assessors and was carried out by a student assistant (MM) and checked for completeness and accuracy by one researcher. Focus group interviews with risk managers and people from the general population were transcribed by an external market research company. The transcripts were double checked by a researcher of the research team (CE) and a research assistant (MM).

S3 File. Focus group interviews – supplement material

Data Analysis

The Kuckartz[1] methodology was used for the qualitative content analysis. A deductive procedure was used in which the codes were defined beforehand on the basis of the respective question guide and applied to the text in order to guarantee the validity and reliability of different coders. In a second step, the category system was further developed during coding on the basis of the text (inductive method) (see page 18 for codesystems).

The qualitative data analysis software *f4analyse* was used for data analysis. The coding and summary of four interviews was done by one of the lead authors (CE). One additional interview was coded by a research assistant (MM) who was not involved in the development of the code system and conducting the interviews. Quotes were added to the summaries of the respective interviews. The researchers on the research team, who were also present during the interviews (CS, AKL, ML), reviewed, revised, and discussed the summaries of the findings based on their field notes. The second lead author (MMD), who was not involved in conducting and evaluating the interviews, also reviewed the findings.

1.1. Focus Group Interview – Risk Assessors

Focus group interviews with risk assessors took place on the 5th and 6th of June 2018 and were carried out in the premises of the BfR. Risk assessors were recruited by the BfR and received no incentives because the interviews took place during working hours.

Participants

Two interviews, one with seven and one with eight participants, were conducted for a duration of two hours. Participants were from various departments of the BfR. In addition to risk assessors from food, product and chemical safety, staff from the communication and legal department joined the interviews.

Interview Guide

A semi-structured interview guide with open questions was used to obtain the opinion of participants on the existing version of the Risk Profile and its individual characteristics (see **Table *1***). In addition, suggestions were sought for improvements to the various dimensions and graphical design.

Table 1: Interview guide Focus group interviews with risk assessors

**Agenda**		**Timeline**
**I**	**Welcome and introduction to the focus group**<br>• Presentation of the risk profile (BfR)<br>• Introduction Harding-Zentrum (Moderation: HC)<br>◦ Short introduction to the project & idea behind focus group<br>◦ Acknowledgement for participation<br>◦ Ethics: Factual anonymization, participation can be cancelled at any time, declaration of consent can be withdrawn for the future, sound recording, signature & declarations of consent<br>◦ Explanation of our roles: Notes and moderation	**15 minutes**

[1] Kuckartz, Udo; Dresing, Thorsten; Rädiker, Stefan & Stefer, Claus. (2008). *Qualitative Evaluation: Der Einstieg in die Praxis*. Wiesbaden: VS Verlag für Sozialwissenschaften.

	• Duration: 2 hours, in which we want to address different aspects<br>• Conversation rules: Do not speak at the same time, let participants finish, no long monologues, balanced participation in the discussion, constructive criticism/comments<br>• Introduction round participants the focus group (work area, experience in risk assessment and with the risk profile) Request for short answers (1-2 sentences)<br>• Clarification of open questions	
**II**	At the beginning, to get into the topic: If you think from the perspective of an actor reading a risk assessment: What do you think are important aspects that should be communicated? Where do you see challenges?	**10 minutes**
**III**	**Discussion of the individual dimensions of the risk profile**<br>→ Hand out profile and project on wall<br>• In your opinion, how can different groups of people be meaningfully classified that differ in their level of risk? How could they best be communicated?<br>o *Optional: Are there any other suggestions?*<br>o *How could these groups be meaningfully divided across the different departments?*<br>• In your opinion, what would be the best way to describe the probability of a risk occurring?<br>o *Optional: Are there any other suggestions?*<br>o *How easy is it to quantify probabilities?*<br>• In your opinion, how can the severity of a health impairment be best communicated?<br>o *Optional: Are there any other suggestions?*<br>o *How easy is it for you to communicate the severity of health impairment? What challenges do you see in this?*<br>o *Do you have suggestions on how to meet these challenges?*<br>• If reliable data for a risk assessment is not available, how do you think this could best be communicated?<br>o *Optional: Are there any other suggestions?*<br>o *How well do the currently existing categories summarize the quality of evidence in the different departments? Is the number of categories too high or too low?*<br>• What possibilities do you see for communicating the controllability of a risk? [We are aware that the subject is sometimes difficult from a legal point of view, BfR has a better overview].<br>o *Optional: Are there any other suggestions?*	**40 minutes (max. 60 minutes)**
**IV**	**Opening of the discussion**<br>• Are there important aspects that you miss in the risk profile?	**40 minutes**

<table>
<tr><td></td><td>

Are there additional dimensions that you would like to see in the profile or that you would find useful?
In which cases can the information from risk assessments not be converted into a risk profile?
One aspect that is crucial to risk assessment is the so-called exposure, i.e. how and by what route a person must come into contact with a potentially harmful substance for an impairment to occur. In your opinion, what is the best way to communicate what exposure is assumed in the risk profile? What categories would be appropriate across the different departments?
Do you see dimensions in the profile that you consider particularly challenging or critical, that could be defined, improved or otherwise changed?

→ And now to come back to the initial question:

Please take the perspective of consumers who would like a summary of the results of the risk assessment.

What information do you think is relevant for consumers but is missing from the risk profiles? [The moderators create a list]
(e.g. benefit, exposure context, dose-response relationship, etc.)
What suggestions do you have for improving the risk profile to make it easier for consumers to understand? (e.g. information or content, layout, categories)

Opinions on verbal analogies for the description of dose-response relationships are sought.

Challenges in communicating dose-response relationships have been mentioned above. The BfR and other institutions have tried in different ways to apply this type of communication to the public.
What do you think of the following examples?

"Single doses of caffeine up to 200 mg - about 3 mg per kilogram body weight (mg / kg bw) do not lead to safety concerns for the general healthy adult population. This corresponds to about 2-3 cups of filter coffee (200 ml)" (EFSA risk assessment coffee)

What do you think are the advantages and disadvantages of this type of communication of risk information? Can you imagine better ways to communicate this type of information?]
[If you had to tell a person [...] who has no experience in risk assessment, how would you explain [...]?
What would help you to communicate [...] to consumers?]

</td><td>(min. 20 minutes)</td></tr>
<tr><td>VI</td><td>
Summary and feedback

Discussion points are summarized and feedback is sought
Are there any points that you consider important in relation to the project but have missed today?
Thank you very much for your participation and have a nice day!

</td><td>10 minutes</td></tr>
</table>

	**End**	**max. 120 minutes**

1.2. Focus Group Interview – Laypeople

Focus groups with laypeople took place on the 3rd and 4th of December 2018 in the premises of a market research company in Berlin. Participants were recruited by another external market research company on behalf of the BfR. Eligible participants were men and women of legal age.

Participants

Two interviews with eight participants each were conducted and lasted for a duration of 90 to 120 minutes. Participants were from the general public. The composition of the focus groups differed somewhat in terms of some socio-economic aspects in order to identify possible inequalities related to the educational qualification, age or mother-tongue speakers. In the first group, most of the participants had completed vocational training (5 out of 8) and their mother tongue was German (7 out of 8). The mean age was 44 years (age range 26 to 58) and sex was equally distributed.

In the second group, three out of eight participants had completed vocational training, four had completed their studies (master or bachelor), one person was studying and the most common mother-tongue speakers were also German (7 out of 8). The mean age was 43 years (age range 23 to 62) and in this group there was an unequal distribution of sex (2 out of 8 were women).

Interview Guide

A semi-structured interview guide with open questions was used to obtain the opinion of the participants from the public on the prototype ideas for individual characteristics of the Risk Profile (see **Table *2***). The guide was developed by the Harding Center in close coordination with the BfR. Questions were asked about comprehensibility, acceptance and applicability of the individual prototype ideas. In addition, suggestions were sought for improvements to the graphical design.

Table 2: Interview guide Focus group interviews with people from the general public

**Agenda**		**Timeline**
**I**	**Welcome and introduction to the focus group**<br><br>• **Welcoming of the participants (BfR)**<br>• **Introduction of the Harding Center and the moderators (BfR)**<br>• **Short introduction to the project & idea behind focus group (HC)**<br><br>I would like to talk to you today about different ways of communicating risks. In cooperation with the German Federal Institute for Risk Assessment (BfR) we have developed various possibilities for illustrating risks, on which we would like to hear your opinion.<br><br>First of all some information about the background of the project:<br><br>The Federal Institute for Risk Assessment (BfR) prepares risk assessments on food, consumer products and chemicals, which are published in the form of scientific	**20 minutes**

<table>
<tr>
<td></td>
<td>
opinions on the BfR homepage, among others. These risk assessments serve as a basis for decisions by various target and interest groups. In addition to the consumers themselves, the central addressees include federal and state ministries, authorities at local, state and federal level as well as consumer associations, scientific institutions and the media. Since 2013, the opinions, which contain detailed explanations on how the BfR conducted the risk assessment, are supplemented with a tabular summary. This tabular summary is called "risk profile". This risk profile is intended to help readers to identify risks at a glance. As a result of an initial evaluation with different target groups, some suggestions for improvement of the risk profile were identified. Together with the BfR, the Harding Center for Risk Literacy is therefore working on improving the risk profile (show empty risk profile briefly).

In today's interview, we would like to hear your opinion on what you, as a consumer, consider to be essential elements in the communication of risks.

At this point I would like to point out that the interview will be recorded with a voice recorder so that we can better evaluate your answers afterwards. My colleague Ms. X will also take notes. The transcript of the audio recording and the notes will be made anonymous, i.e. we will remove all references to your identity and summarize the answers in such a way that no conclusions can be drawn about your person. The audio file and your declaration of consent are kept separately from each other without access by outsiders. You can withdraw your consent at any time (even after the interview). (get declaration of consent signed; the audio recording will only start after the round of interviews). The audio file will be deleted after transcription.

Warming up: Before we start with the interview, I would like to do a small round of introductions so that we all get to know each other briefly.
I would like to ask you to introduce yourself briefly and tell us whether you have ever heard of the work of the Federal Institute for Risk Assessment. (1-2 sentences)
Thank you very much. During the next two hours, we will ask you some questions regarding the communication of risks. In order to do so, we will show you some examples that we have developed in this context.
Conversation rules: Do not speak at the same time, let participants finish, balanced participation in the discussion, constructive criticism/comments
Before we start the interview: Are there any open questions?
Then I will start the recording now

</td>
<td></td>
</tr>
<tr>
<td>II</td>
<td>Information needs with regard to everyday risks

What do you think is important information regarding risks, e.g. in the area of food safety, that should be communicated?
Do you actively seek information on the safety of specific foods or products and if so, where?

</td>
<td>15-20 minutes</td>
</tr>
</table>

<table>
<tr><td></td><td>

How would you like to be informed about risks in food or everyday products? For example, there is oral or written information. Do you prefer text for reading or graphics that illustrate the risk?
In your opinion, what are suitable media for communicating health risks (e.g. television, radio, Internet, etc.)?

</td><td></td></tr>
<tr><td>III</td><td>
Brief presentation of current risk characteristics and evaluation of risk profile prototypes for individual dimensions
Now I would like to show you some possibilities how different aspects, which are necessary for the risk assessment, could be presented. I would like to ask you to give me feedback on this.

Communication of the facts
First of all, I would like to show you a possibility how the facts could be presented.
→ slide 3
Questions to the participants
On example A:
How do you rate this selection of symbols to graphically represent the situation?
On example B:
How do you rate these presentations for communicating the facts?
Does this presentation contain too little or too much information? (optional: what would you add?)
How understandable do you find this type of presentation?
How useful do you find this type of presentation?
On example A+B:
What kind of presentation do you prefer?
What would you wish in terms of communicating the facts?
Do you have a suggestion on how this aspect could be better presented?
Communicating about the affected group
The risk of a health impairment depends first of all on who is affected. There are risks that affect the entire population. However, there are also risks from certain products or substances that only affect certain groups of people (e.g. children, pregnant women)
→ slide 4 (+5) with prototypes
Questions to the participants
On example B:
How understandable do you find this kind of presentation?
How useful do you find this type of presentation?

</td><td>60 minutes</td></tr>
</table>

What kind of presentation do you prefer?

What would you wish in terms of communication about the affected group?

Do you have a suggestion on how this aspect could be better presented?

- **Communicating about the possible contact with a product or substance:**

Harmful substances from food or products can be absorbed by humans via various pathways. For example, contact is possible through consumption or through the skin.

→ slide 6

Questions to the participants

On example B:

How understandable do you find this kind of presentation?

How useful do you find this type of presentation?

What kind of presentation do you prefer?

What would you wish in terms of communicating about the possible contact with a product or substance?

Do you have a suggestion on how this aspect could be better presented?

- **Communicating about the severity of possible health impairments in case of contact:**

Another aspect of risk communication is the severity of health impairments in case of contact with a substance or product: for instance, does only mild nausea occur or do stronger symptoms, such as severe nausea, occur?

→ slide 7 with prototypes

Questions to the participants

On example B:

How understandable do you find this kind of presentation?

How useful do you find this type of presentation?

What kind of presentation do you prefer?

What would you wish in terms of communicating about the severity of a health impairment in case of contact?

Do you have a suggestion on how this aspect could be better presented?

Optional:

What kind of presentation would you like to see if several types of health damage can occur?

- **Communicating about the probability of health impairment in case of contact:**

  This aspect describes the likelihood of a health impairment in case of contact with a potentially harmful substance or a potentially harmful product. This is because impairment does not always occur. For example, how likely is a health risk if you eat raw or underheated eggs or egg dishes in which salmonella has been detected?

  → slide 8 with prototypes

  Questions to the participants

  On example B:

  How understandable do you find this kind of presentation?

  How useful do you find this type of presentation?

  What kind of presentation do you prefer?

  What would you wish in terms of communicating about the probability of a health impairment in case of contact?

  Do you have a suggestion on how this aspect could be better presented?

- **Communicating about the combination of severity and probability of a health impairment**

  In the following we have combined the probability and severity of a health impairment.

  → slide 9 with prototypes

  Questions to the participants

  How understandable do you find the combined presentation of severity and probability of a health impairment?

  How useful do you find this type of combined presentation?

  Do you have a suggestion on how this aspect could be better presented?

- **Communicating about the reliability of the data:**

  This aspect deals with the reliability of the data on the basis of which a risk assessment was carried out. For example, are there any studies at all that have been carried out with human participants or are there possibly only studies on animals or individual human cells?

  → slide 10 with prototypes

  Questions to the participants

  On example B:

  How understandable do you find this kind of presentation?

  How useful do you find this type of presentation?

	What kind of presentation do you prefer?<br><br>What would you wish in terms of communicating about the reliability of the data?<br><br>Do you have a suggestion on how this aspect could be better presented?<br><br>• **Communicating about the controllability**<br><br>This aspect deals with how to control possible health damages to consumers or through risk management.<br><br>How can consumers protect themselves from health damage? One possibility would be, for example, to avoid certain ingredients in products, to enjoy a certain food only moderately or to cook it thoroughly.<br><br>In many cases, however, risk management is also required, which can make recommendations to politicians or industry. This raises the question: What control options does risk management have?<br><br>→ slide 11 with prototypes<br><br>**Questions to the participants**<br><br>How understandable do you find this kind of presentation?<br><br>How useful do you find this type of presentation?<br><br>What kind of presentation do you prefer?<br><br>What would you wish in terms of communicating about the controllability?<br><br>Do you have a suggestion on how this aspect could be better presented?<br><br>• **Communicating about limit values**<br><br>Limit values are often set in the risk assessment. In many cases, it is assumed that a substance is not dangerous for humans as long as the limit value is not exceeded. One way of defining such a limit value is to look at the amount of the substance that people need to ingest in order to observe first adverse effects (e.g. abdominal pain, heart palpitation). A safety margin for this value is then chosen and a lower value is defined as the limit value. Here, this is illustrated by the example of magnesium as a food supplement.<br><br>→ slide 14<br><br>**Questions to the participants**<br><br>How understandable do you find this kind of presentation?<br><br>How useful do you find this type of presentation?<br><br>Do you have a suggestion on how this aspect could be better presented?	
**IV**	**General**<br><br>• When thinking about the information discussed, do you find there are relevant information missing? If so, what are you missing?	**max. 10 minutes**

	• Which information could you have done without? Was there information that were not interesting to you?<br>**Opinions on verbal analogies for the description of dose-response relationships are sought.**<br>• One challenge in risk communication is the communication of dose-response relationships, i.e. that the effect of a substance depends on the consumed dose. The BfR and other institutions have tried in different ways to apply this type of communication to the public.<br>• What do think of the following example?<br>"The intake of up to 200 mg of caffeine within a short period of time do not lead to health concerns for the general healthy adult population. This corresponds to about two cups of filter coffee (200 ml). Over the course of the day, adults can drink about twice as much, i.e. 400 mg. This corresponds to about four to five cups of filter coffee.<br>In general, 3 milligrams of caffeine per kilogram of body weight as a single dose and 5.7 milligrams spread over the day are not problematic for adults. This means that an adult with a body weight of 65 kg can take up about 195 mg of caffeine as a single dose without having to fear health consequences. A cup of filter coffee of 200 ml contains about 90 mg of caffeine."	
**V**	**Summary and feedback**<br>Now I would like to briefly summarize the key points that we have discussed today.<br>• Are there any points that you consider important in relation to the project but have missed today?<br>• Thank you very much for your participation and have a nice day!	**10 minutes**
	**End**	**max. 120 minutes**

1.3. Focus Group Interview – Risk Managers

A two-hour interview with eight participants from risk management was conducted on the 3rd of December 2018 in the premises of a market research company in Berlin.

Participants

Interview participants were risk managers, for instance, those who work with the risk assessments of the BfR to derive decisions on risk management for consumers.

Interview Guide

A semi-structured interview guide with open questions was used to obtain the opinion of the risk managers on the prototype ideas for individual characteristics of the Risk Profile (see **Table *3***). The guide was developed by the Harding Center in close coordination with the BfR. Questions were asked about comprehensibility, acceptance and applicability of the individual prototype ideas. In addition, suggestions were sought for improvements to the graphical design.

S3 File. Focus group interviews – supplement material

Table 3: Interview guide Focus group interviews with risk managers

**Agenda**		**Timeline**
**I**	**Welcome and introduction to the focus group**<br>• **Welcoming of the participants (BfR)**<br>• **Introduction of the moderators (BfR)**<br>• **Joint project of the BfR and the Harding Center for Risk Literacy (HC) (BfR)**<br>• **Introduction of the Harding Center (HC)**<br>Hello and welcome, my name is X, and I am a research scientist at the Harding Center for Risk Literacy.<br>We are part of the Max Planck Institute for Human Development and examine, for example, the question of which skills, but also which instruments and types of representations of information can help people to engage with risks in a competent way and to make informed decisions.<br>I will lead this focus group interview. The aim of this interview is to get feedback from you regarding various ideas for illustrating risks in the context of risk assessments. We would also like to learn more about your needs or wishes as risk managers regarding the presentation of information in risk assessments by BfR.<br><br>At this point I would like to point out that the interview will be recorded with a voice recorder so that we can better evaluate your answers afterwards. My colleague Ms. X will also take notes. The transcript of the audio recording and the notes will be made anonymous, i.e. we will remove all references to your identity and summarize the answers in such a way that no conclusions can be drawn about your person. The audio file and your declaration of consent are kept separately from each other without access by outsiders. You can withdraw your consent at any time (even after the interview). (get declaration of consent signed; the audio recording will only start after the round of interviews). The audio file will be deleted after transcription.<br><br>• **Warming up**: Before we start with the interview, I would like to do a small round of introductions so that we all get to know each other briefly.<br>o I would like to ask you to introduce yourself briefly, to name your current field of work, and to briefly explain, whether, and if so, to what extent, you are working with the BfR risk assessments.<br>o Are you familiar with the BfR risk profile?<br>• Thank you very much. During the next two hours, we will ask you some questions regarding the communication of risks. In order to do so, we will show you some examples that we have developed in this context.	**20**<br><br>**minutes**

	• Conversation rules: Do not speak at the same time, let participants finish, balanced participation in the discussion, constructive criticism/comments<br>• Before we start the interview: Are there any open questions?<br>• Then I will start the recording now	
**II**	**General**<br>• (If you haven't stated this yet) Since when do you work in the field of risk managment? What exactly is your field of work?<br>• As risk managers, what challenges do you face when using risk information for your work?<br>***Optional:***<br>o To what extent could a better presentation of the information contribute to an improvement?<br>• What information is relevant for your work? Are there any information or aspects that you miss in the risk information available to you?<br>• If you now think of a summary of the contents of risk information and of your work: What information should be included in such a summary and how should it be presented?	**30 minutes**
**III**	**Brief presentation of current risk characteristics and evaluation of risk profile prototypes for individual dimensions**<br>Now I would like to show you some possibilities how different aspects, which are necessary for the risk assessment, could be presented.<br>• **Communication of the facts**<br>First of all, I would like to show you a possibility how the facts could be presented.<br>→ slide 3<br>**Questions to the participants**<br>***On example A:***<br>How do you rate this selection of symbols to graphically represent the situation?<br>***On example B:***<br>How do you rate these presentations for communicating the facts?<br>Does this presentation contain too little or too much information? (optional: what would you add?)<br>How understandable do you find this type of presentation?<br>How useful do you find this type of presentation?<br>***On example A+B:***<br>What kind of presentation do you prefer?<br>What would you wish in terms of communicating the facts?	**max. 50 minutes**

Do you have a suggestion on how this aspect could be better presented?

- **Communicating about the affected group**
The risk of a health impairment depends first of all on who is affected. There are risks that affect the entire population. However, there are also risks from certain products or substances that only affect certain groups of people (e.g. children, pregnant women)

→ slide 4 (+5) with prototypes

Questions to the participants

On example B:

How understandable do you find this kind of presentation?

How useful do you find this type of presentation?

What kind of presentation do you prefer?

What would you wish in terms of communication about the affected group?

Do you have a suggestion on how this aspect could be better presented?

- **Communicating about the possible contact with a product or substance:**

Harmful substances from food or products can be absorbed by humans via various pathways. For example, contact is possible through consumption or through the skin.

→ slide 6

Questions to the participants

On example B:

How understandable do you find this kind of presentation?

How useful do you find this type of presentation?

What kind of presentation do you prefer?

What would you wish in terms of communicating about the possible contact with a product or substance?

Do you have a suggestion on how this aspect could be better presented?

- **Communicating about the severity of possible health impairments in case of contact:**

Another aspect of risk communication is the severity of health impairments in case of contact with a substance or product: for instance, does only mild nausea occur or do stronger symptoms, such as severe nausea, occur?

→ slide 7 with prototypes

Questions to the participants

On example B:

How understandable do you find this kind of presentation?

How useful do you find this type of presentation?

What kind of presentation do you prefer?

What would you wish in terms of communicating about the severity of a health impairment in case of contact?

Do you have a suggestion on how this aspect could be better presented?

Optional:

What kind of presentation would you like to see if several types of health damage can occur?

- **Communicating about the probability of health impairment in case of contact:**

  Another question arises: How likely is it that a person who has come into contact with a substance or product will suffer health damage? A closely related question is also: How likely is it that a person will come into contact with a harmful substance?

  → slide 8 with prototypes

  Questions to the participants

  On example B:

  How understandable do you find this kind of presentation?

  How useful do you find this type of presentation?

  What kind of presentation do you prefer?

  Do you have a suggestion on how this aspect could be better presented?

- **Communicating about the combination of severity and probability of a health impairment**

  In the following we have combined the probability and severity of a health impairment.

  → slide 9 with prototypes

  Questions to the participants

  How understandable do you find the combined presentation of severity and probability of a health impairment?

  How useful do you find this type of combined presentation?

  Do you have a suggestion on how this aspect could be better presented?

- **Communicating about the reliability of the data:**

  This aspect deals with the reliability of the data on the basis of which a risk assessment was carried out. For example, are there any studies at all that have been

carried out with human participants or are there possibly only studies on animals or individual human cells?

→ slide 10 with prototypes

Questions to the participants

On example B:

How understandable do you find this kind of presentation?

How useful do you find this type of presentation?

What kind of presentation do you prefer?

What would you wish in terms of communicating about the reliability of the data?

Do you have a suggestion on how this aspect could be better presented?

- **Communicating about the controllability**

This aspect deals with how to control possible health impairments to consumers or through risk management.

How can consumers protect themselves from health impairment? One possibility would be, for example, to avoid certain ingredients in products, to enjoy a certain food only moderately or to cook it thoroughly.

In many cases, however, risk management is also required, which can make recommendations to politicians or industry. This raises the question: What control options does risk management have?

→ slide 11 with prototypes

Questions to the participants

How understandable do you find this kind of presentation?

How useful do you find this type of presentation?

What kind of presentation do you prefer?

Do you have a suggestion on how this aspect could be better presented?

- **Communicating about limit values**

Limit values are often set in the risk assessment. In many cases, it is assumed that a substance is not dangerous for humans as long as the limit value is not exceeded. One way of defining such a limit value is to look at the amount of the substance that people need to ingest in order to observe first adverse effects (e.g. abdominal pain, heart palpitation). A safety margin for this value is then chosen and a lower value is defined as the limit value. Here, this is illustrated by the example of magnesium as a food supplement.

→ slide 13

Questions to the participants

	How understandable do you find this kind of presentation?<br>How useful do you find this type of presentation?<br>What would you call the range between the LOAEL (if necessary, briefly explain what this is) and the limit value?<br>What do you think of the following suggestions for what this range might be called?<br>→ slides 14–16<br>Do you have a suggestion on how this aspect could be better presented?	
**IV**	**General**<br>• When thinking about the information discussed, do you find there are relevant information missing? If so, what are you missing?<br>• Which information could you have done without? Was there information that were not interesting to you?<br>**Opinions on verbal analogies for the description of dose-response relationships are sought.**<br>• One challenge in risk communication is the communication of dose-response relationships, i.e. that the effect of a substance depends on the consumed dose. The BfR and other institutions have tried in different ways to apply this type of communication to the public.<br>• What do think of the following example?<br>"The intake of up to 200 mg of caffeine within a short period of time do not lead to health concerns for the general healthy adult population. This corresponds to about two cups of filter coffee (200 ml). Over the course of the day, adults can drink about twice as much, i.e. 400 mg. This corresponds to about four to five cups of filter coffee.<br>In general, 3 milligrams of caffeine per kilogram of body weight as a single dose and 5.7 milligrams spread over the day are not problematic for adults. This means that an adult with a body weight of 65 kg can take up about 195 mg of caffeine as a single dose without having to fear health consequences. A cup of filter coffee of 200 ml contains about 90 mg of caffeine."	**max. 10 minutes**
**V**	**Summary and feedback**<br>Now I would like to briefly summarize the key points that we have discussed today.<br>• Are there any points that you consider important in relation to the project but have missed today?<br>• Thank you very much for your participation and have a nice day!	**10 minutes**
**VI**	**End**	**max. 120 minutes**

2. Codesystems

Codesystem risk assessor – Interviews

Category	Code	Subcode	No	Explanation/Classification
1. Background information	1.1 Interview date		1.	Interview 5.6.18/Interview 6.6.18
	1.2 Working area		2.	Working area within the BfR: risk assessment/other
	1.3 Risk Profile familiarity		3.	Filled out/Known/Not known
2. General	2.1 Relevant aspects		4.	Important aspects that should be communicated
	2.2 Challenges		5.	Challenges in risk communication
3.	3.1 Affected group	3.1.1 Classification	6.	How can different groups of people be meaningfully classified who differ in their vulnerability? What would be the best way to communicate them?
	3.2 Probability		7.	What is the best way to describe the probability of a risk occurring?
	3.3 Severity	3.3.1 Characteristics	8.	Evaluation of characteristics
		3.3.2 Challenges	9.	Challenges in severity communication
		3.3.3 Suggestions	10.	Suggestions to improve severity communication
	3.4 Certainty of data	3.4.1 Characteristics	11.	Evaluation of characteristics
		3.4.2 Challenges	12.	Challenges in communicating certainty of data
		3.4.3 Suggestions	13.	Suggestions to improve communication about certainty of data
	3.5 Controllability	3.5.1 Characteristics	14.	Evaluation of characteristics
		3.5.2 Challenges	15.	Challenges in communicating controllability
		3.5.3 Suggestions	16.	Suggestions to improve communicating controllability
	3.6 Whole profile	3.6.1 Target group	17.	Target group of the Risk Profile: general population/risk manager/media
		3.6.2 Layout	18.	Evaluation of the layout: is liked/not liked
		3.6.3 Dimensions	19.	Additional dimensions that participants would find useful: Yes/No; What dimensions?
		3.6.4 Aspects	20.	Aspects that participants are missing: Yes/No; What aspects?
		3.6.5 Challenges	21.	Challenges concerning the whole profile
		3.6.6 Suggestions	22.	Suggestions for improvement of the whole Risk Profile

**4. Additions**	4.1 Limitations		23.	Cases in which information from the risk assessments can not be transformed into a Risk Profile: Yes/No; What kind of information?
	4.2 Exposure context			
		4.2.1 Suggestions	24.	Best way to communicate the exposure in the Risk Profile?; Which categories would suit all departments?
	4.3 Verbal analogies		25.	Evaluation of examples on the dose-response relationship

Codesystem lay population – Interviews

Category	Code	Subcode	No	Explanation/Classification
**1. Background information**	1.1 Interview date		1.	Interview 5.6.18/Interview 6.6.18
	1.2 Familiarity		2.	BfR known/not known
**2. Information needs and preferences in the context of everyday risks**	2.1 Information needs		3.	Important information concerning (food) risks that should be communicated
	2.2 Search for information		4.	Search for information on the safety of certain foods or products and sources for searching
	2.3 Information preferences		5.	Preferences about information provision (e.g., oral or written information, text or visualization)
	2.4 Information channels		6.	Appropriate channels for the communication of risks
**3. Evaluation of Risk Profile (aspects)**	3.1 Facts	3.1.1 Visualisation	7.	Evaluation of visualisation
		3.1.2 Prototype	8.	Prototype preference
		3.1.3 Comprehensiblity	9.	Comprehensibility of prototypes and content
		3.1.4 Suggestions	10.	Suggestions to improve communication about the facts
	3.2 Affected group	3.2.1 Visualisation	11.	Evaluation of visualisation
		3.2.1 Prototype	12.	Prototype preference
		3.2.3 Comprehensiblity	13.	Comprehensibility of prototypes and content
		3.2.4 Suggestions	14.	Suggestions to improve communication about the affected groups
	3.3 Exposure	3.3.1 Visualisation	15.	Evaluation of visualisation
		3.3.1 Prototype	16.	Prototype preference
		3..3 Comprehensiblity	17.	Comprehensibility of prototypes and content
		3.3.4 Suggestions	18.	Suggestions to improve communication about the exposure context

	3.4 Severity	3.4.1 Visualisation	19.	Evaluation of visualisation
		3.4.2 Prototype	20.	Prototype preference
		3.4.3 Comprehensibility	21.	Comprehensibility of prototypes and content
		3.4.4 Suggestions	22.	Suggestions to improve severity communication
	3.5 Probability	3.5.1 Visualisation	23.	Evaluation of visualisation
		3.5.2 Prototype	24.	Prototype preference
		3.5.3 Comprehensibility	25.	Comprehensibility of prototypes and content
		3.5.4 Suggestions	26.	Suggestions to improve probabilty communication
	3.6 Severity and Probability	3.6.1 Visualisation	27.	Evaluation of visualisation
		3.6.2 Prototype	28.	Prototype preference
		3.6.3 Comprehensibility	29.	Comprehensibility of prototypes and content
		3.6.4 Suggestions	30.	Suggestions to improve severity and probabilty communication
	3.7 Certainty of data	3.7.1 Characteristics	31.	Evaluation of visualisation
		3.7.2 Challenges	32.	Prototype preference
		3.7.3 Comprehensibility	33.	Comprehensibility of prototypes and content
		3.7.4 Suggestions	34.	Suggestions to improve communication about certainty of data
	3.8 Controllability	3.8.1 Visualisation	35.	Evaluation of characteristics
		3.8.2 Prototype	36.	Prototype preference
		3.8.3 Comprehensibility	37.	Comprehensibility of prototypes and content
		3.8.4 Suggestions	38.	Suggestions to improve communicating of controllability
	3.9 Thresholds	3.9.1 Visualisation	39.	Evaluation of characteristics
		3.9.2 Prototype	40.	Prototype preference
		3.9.3 Comprehensibility	41.	Comprehensibility of prototypes and content
		3.9.4 Suggestions	42.	Suggestions to improve communicating of thresholds
**4. Gerenal**	4.1 Information content		43.	Lack of relevant information/Information to reduce
	4.2 Verbal analogies		44.	Evaluation of examples on the dose-response relationship
	4.3 Other comments		45.	

Codesystem risk manager - Interview

Category	Code	Subcode	No	Explanation/Classification
1. Background information	1.1 Interview date		1	Interview 3.12.18
	1.2 Working area		2	Current field of work within risk management
	1.3 Work with risk assessments of BfR		3	No/Yes
	1.4 Risk Profile familiarity		4	Known/Not known
2. General	2.1 Working in the field of risk management		5	Working in the field of risk management and current field of work
	2.2 Challenges		6	Challenges in the use of risk information
	2.3 Information needs for work		7	Relevant information for own work
	2.4 Missing information		8	Missing information/aspects in available risk information
	2.5 Expectations on summary information presentation		9	Expectations on information summary and representation
3. Evaluation of Risk Profile (aspects)	3.1 Facts	3.1.1 Visualisation	10	Evaluation of visualisation
		3.1.2 Prototype	11	Prototype preference
		3.1.3 Comprehensiblity	12	Comprehensibility of prototypes and content
		3.1.4 Suggestions	13	Suggestions to improve communication about the facts
	3.2 Affected group	3.2.1 Visualisation	14	Evaluation of visualisation
		3.2.2 Prototype	15	Prototype preference
		3.2.3 Comprehensiblity	16	Comprehensibility of prototypes and content
		3.3.4 Suggestions	17	Suggestions to improve communication about the affected groups
	3.3 Exposure	3.3.1 Visualisation	18	Evaluation of visualisation
		3.3.2 Prototype	19	Prototype preference
		3.3.3 Comprehensiblity	20	Comprehensibility of prototypes and content

		3.3.4 Suggestions	21	Suggestions to improve communication about the exposure context
	3.4 Severity	3.4.1 Visualisation	22	Evaluation of visualisation
		3.4.2 Prototype	23	Prototype preference
		3.4.3 Comprehensibility	24	Comprehensibility of prototypes and content
		3.4.4 Suggestions	25	Suggestions to improve severity communication
	3.5 Probability	3.5.1 Visualisation	26	Evaluation of visualisation
		3.5.2 Prototype	27	Prototype preference
		3.5.3 Comprehensibility	28	Comprehensibility of prototypes and content
		3.5.4 Suggestions	29	Suggestions to improve probabilty communication
	3.6 Severity and Probability	3.6.1 Visualisation	30	Evaluation of visualisation
		3.6.2 Prototype	31	Prototype preference
		3.6.3 Comprehensibility	32	Comprehensibility of prototypes and content
		3.6.4 Suggestions	33	Suggestions to improve severity and probabilty communication
	3.7 Certainty of data	3.7.1 Characteristics	34	Evaluation of visualisation
		3.7.2 Challenges	35	Prototype preference
		3.7.3 Comprehensibility	36	Comprehensibility of prototypes and content
		3.7.4 Suggestions	37	Suggestions to improve communication about certainty of data
	3.8 Controllability	3.8.1 Visualisation	38	Evaluation of characteristics
		3.8.2 Prototype	39	Prototype preference
		3.8.3 Comprehensibility	40	Comprehensibility of prototypes and content
		3.8.4 Suggestions	41	Suggestions to improve communicating of controllability
	3.9 Thresholds	3.9.1 Visualisation	42	Evaluation of characteristics
		3.9.2 Prototype	43	Prototype preference
		3.9.3 Comprehensibility	44	Comprehensibility of prototypes and content
		3.9.4 Suggestions	45	Suggestions to improve communicating of thresholds
**4. General**	4.1 Information content		46	Lack of relevant information/Information to reduce
	4.2 Verbal analogies		47	Evaluation of examples on the dose-response relationship
	4.3 Other comments		48	
