# Supplementary material for: Identifying content to improve risk assessment communications within the Risk Profile: Literature reviews and focus groups with expert and non-expert stakeholders
Source: PLoS One. 2022 Apr 11;17(4):e0266800. doi: 10.1371/journal.pone.0266800 (PMC9000125; doi:10.1371/journal.pone.0266800)
Supplement: Attachment — Submitted filename: Response to Reviewers.pdf [file pone.0266800.s004.pdf]

We thank the Editor Dr. Tim Mathes for taking the time to review a previous version of our manuscript. On review of the comments, we note that many of these comments can be easily addressed or the information can be found within the manuscript or in the Supplementary material. We provide responses to each comment below, having addressed each of these points.

- Editor: The approach or methods for initial search step are not described.

Our initial search step involved searching for existing examples of risk communication tools/strategies used by other risk assessment institutions. We did not include much detail in the manuscript as the search turned up only one other example (described in the manuscript) and we therefore elected to follow the approach used to develop evidence-based health information: focus the strategy on evidence for relevant endpoints and obtain information on the needs and requirements from target groups to develop prototypes. Nevertheless, we have provided more detail on the initial search for existing examples and the approach that was taken in the Methods.

"A search for the risk communication strategies of risk assessment institutions in Europe, as well as international and European risk assessment agencies (e.g., European Food Safety Authority, The European Food Information Council) found that almost all institutions used scientific articles or reports to publish their risk assessments, and none used a standardized approach to communicate results of individual risk assessments to lay audiences (with some exceptions for specific risk assessments of public interest [5] or general information or broad overviews of risk assessments [21])."

- Editor: I is not clear were the systematic review process has been rapid, i.e. what and where shortcuts were made, and what is the rational.

In the methods, we describe in detail our search procedure and the way in which we defined our rapid review strategy: ***" Like systematic reviews, rapid reviews are part of the knowledge synthesis family [23] where "processes are accelerated and methods are streamlined to complete the review more quickly than is the case for typical systematic reviews" [24](p3). Rapid reviews are employed to provide an overview of the latest evidence on a specific topic to support decision-making while ensuring that the scientific imperative of methodological rigor is met [23, 24]. At the time we conducted the rapid reviews, there was no uniform method for conducting rapid reviews. Nevertheless, the methodological steps we took adhere to all recommendations outlined in the recently published Cochrane Rapid Reviews Methods group guidelines for conducting rapid reviews (with the single exception that the protocol was not published online) [23]. We describe our methods in detail in the Supplementary Material (see S1 File)."***

Specifically, we restricted our search in the following way:

- Used a limited number of databases between 2 and 4, which varied according to the search (databases used were for instance: PubMed, PsycINFO, Scopus and Web of Science),

- Restricted the types of studies (English-language, last 5 or 10 years only for most of the searches),

- Limited search for grey literature to a search of reference lists and relevant journals,

- Limitation of double-checking during study selection procedure (30% of a random sample was double-checked).

We note potential limitations to the rapid review in the limitation section: e.g., that we may have missed relevant material by restricting our search (e.g., by search time period, number of databases, study design) and focusing only on high quality studies (e.g., systematic reviews, RCTs).

- Editor: It is quite unusual to synthesizes secondary (systematic reviews) and primary studies (RCTs), unless existing systematic reviews are out-dated (e.g. with new RCTs) and the primary studies are only used to supplement the systematic reviews.

We agree with the editor’s comment and would like to clarify that our approach is consistent with this strategy. First, rapid reviews served to generate an overview of existing approaches to communicating relevant aspects of risk assessment results to generate ideas for the revision of the risk profile.

We would like to emphasise that the purpose of these reviews was not to integrate findings into a single review; rather the searches were independent and focused on identifying communication strategies for each of the individual dimensions (e.g., communicating uncertainty, visualizing threshholds). This is consistent with an evidence-based approach and was also applied in the process of creating the German guideline on evidence-based patient information (Lühnen, J., Albrecht, M., Hanßen, K., Hildebrandt, J., & Steckelberg, A. (2015). Guideline for the development of evidence-based patient information: insights into the methods and implementation of evidence-based health information. *Zeitschrift für Evidenz, Fortbildung und Qualität im Gesundheitswesen*, *109*(2), 159-165). In this regard, a combined search strategy was far too complex and our initial attempt to combine the search strategies led to an unmanageable number of hits with very inappropriate results. Therefore, the searches were split in order to be able to search in a more targeted way. Therefore, while one search summarizes evidence based on individual studies because there is not much literature on the topic (search on "How to communicate the severity of health impairments), others were based on findings from systematic reviews where there is a broader evidence basis. The findings from the individual searches were summarized for the purpose of informing tool development.

In Supplementary material S1, we describe that our focus was on systematic reviews, with or without meta-analyses, and primary studies, with the focus initially on systematic reviews for each topic. We would like to clarify that systematic reviews were prioritised, with RCTs supporting or supplementing these reviews where they were outdated or were no Systematic Reviews were available. Only for the uncertainty search and the search on "How to communicate the severity of health impairments" no systematic reviews could be identified and primary studies respectively, primary studies with findings from literature reviews were used.

We added this detail to the Methods and to the Supplementary material.

- Editor: The literature search is technical flawed. Relevant MeSH-terms and keywords are missing.

We included the following additional detail on how we identified search terms in the S1 supplement file to provide clarity for the readers: ***“We took a comprehensive approach to identify and revise relevant search terms. First, we dedicated multiple joint research meetings to identify relevant search terms and further refined these in several meetings with a dedicated information specialist from the library of the Max Planck institute for Human Development. These terms were iteratively adjusted to refine the search strategies multiple times. The final search terms excluded a number of initially promising***

terms because the hits were completely off topic and drastically increased the proportion of irrelevant studies (these terms were excluded after repeated reviews of search result samples)."

- Editor: AMSTAR (1) was used although AMSTAR 2 already existed at the time of the assessment. In addition, it is not adequate to calculate an overall score for AMSTAR.

The editor is correct that AMSTAR 2 already existed at the time of our assessment, and we made a conscious decision not to use AMSTAR 2 because, unlike AMSTAR 1, AMSTAR 2 was not validated at this time. This is also mentioned in the current Cochrane manual "Evaluation of systematic reviews Version 1.0" (Cochrane Deutschland, Arbeitsgemeinschaft der Wissenschaftlichen Medizinischen Fachgesellschaften – Institut für Medizinisches Wissensmanagement. „Bewertung von systematischen Übersichtsarbeiten: ein Manual für die Leitlinienerstellung“. 1. Auflage 2017. Available: Cochrane Deutschland: http://www.cochrane.de/de/review-bewertung-manual). We added information for our rationale for using AMSTAR 1 to the methods.

It is also correct that a sum score for an overall assessment at AMSTAR 1 was not intended in the first AMSTAR development paper. However, at the time the literature searches were carried out, this was still being discussed, as a sum score was also reported in the validation paper of AMSTAR 1. We therefore decided to report one at that time. In order to take into account the recent consensus on this, we would add statements about the individual limitations of the included systematic reviews in the supplement S1 instead of an overall assessment, if necessary.

- Editor: Only experts from one institution were included in the focus groups.

As described in our manuscript, several focus group interviews were conducted with different user groups. In addition to the literature search for identifying key elements for communicating risk communication aspects, initially focus group interviews were conducted with risk assessors who are solely responsible for preparing risk assessments and risk profiles in Germany. The aim was to identify barriers to communicating risk assessments, gather feedback on the 2013 risk profile, and develop ideas for a new risk profile from those who are directly involved in the risk assessment communication process.

The results of the literature reviews and focus group interviews with risk assessors led to the development of several prototypes for each dimension of the risk profile (V1). Subsequently, focus groups were conducted with different target audiences (risk managers from various fields of risk management, and the general public) to identify information needs and evaluate the prototypes for each dimension of the risk profile. The results of the focus groups with risk managers and lay people were also incorporated into the development of a risk profile redesign. The redesign of the risk profile was then tested in a user testing with risk assessors from the first focus group interviews, as they are the ones who have to complete it in the end.
- Editor: The sampling strategy and sample characteristics of the population are not described and thus it cannot be assessed if the sample is representative.

We conducted focus groups with three distinct user groups: national risk assessors, risk managers, and the general public. The BfR (German Federal Institute for Risk Assessment) has the statutory task in Germany to assess and communicate risk assessment results. They served as the target user-group and the profile design was developed with the goal to be implemented in this institution. As such, the participants from this institution were the relevant target and user-group, and we made sure to include a diverse sample of individuals from different departments to ensure diversity of

opinions, needs, and feedback. We also conducted focus groups with other potential user groups, including risk managers (from different institutions) and members of the general public.

We addressed the editors comment in our limitation section, and make explicit that the transferability of the risk profile for use in other institutions should be assessed in future research. We also already address other potential limitations to our samples in the discussion.

Regarding the recruitment process, we provide details for each of the focus groups samples. For the risk assessors and risk managers, owing to the small number of participants within specific institutions, we could not provide detailed socio-demographic information while still ensuring data protection. Nevertheless, we have endeavored to include additional detail about the composition of the sample where possible:

For the focus groups with risk assessors, we report on page 7: "*Individuals from all scientific departments at the BfR were invited to participate on a voluntary basis. The focus groups included participants with and without previous experience working with the 2013 Risk Profile. Participants were primarily risk assessors from various departments of the BfR (n=13), and included members of the press and legal units who were involved in communicating about risk assessments or the legal aspects of BfR risk communications (n=2). Due to the small number of participants, differentiated information on the fields of work and demographics is not reported in order to ensure data protection*."

For the risk managers, we report on page 7: *"Participants (n =8) from the field of risk management were recruited from German ministries and federal offices by the BfR. Risk managers from these agencies use the results of risk assessments from the BfR to make decisions about risk management for consumers (e.g., determine limit values for certain substances in food; withdraw products from the market). Due to the small number of participants, differentiated information on the fields of work and demographics is not reported in order to ensure data protection."*

We will add the information about the gender and the rough affiliation of the participants from the field of risk management, as well as the recruitment process: ***"Three men and five women were recruited, with five participants from the BVL (Federal Office of Consumer Protection and Food Safety) and three participants from the BMEL (Federal Ministry of Food and Agriculture). Individuals were nominated by the two institutions. BfR had sent a letter to the presidents of the two partner institutions describing the project and the objective of the focus groups and requesting that the organizations nominate individuals from as many different fields of work as possible who regularly come into contact with risk assessments / risk profiles. After receiving the contact details of suitable persons, information for the appointment was sent. A selection was thus made by the two partner authorities."***

For the general public, we report on pages 7-8: "*The participants were recruited by an external market research company with the goal to recruit individuals from various backgrounds (e.g., age, education)… In the first group (n=8), the majority of participants had completed vocational training (n=5) and their mother tongue was German (n=7). The mean age was 44 years (age range 26-58 years) with equal gender representation. In the second group (n=8), three participants had completed vocational training, four had completed their studies (master or bachelor), one participant was currently studying and most reported German as their mother tongue (n=7). The mean age was 43 years (age range 23-62 years) and two participants were women.*"
