# Supplementary material for: Identifying content to improve risk assessment communications within the Risk Profile: Literature reviews and focus groups with expert and non-expert stakeholders
Source: PLoS One. 2022 Apr 11;17(4):e0266800. doi: 10.1371/journal.pone.0266800 (PMC9000125; doi:10.1371/journal.pone.0266800)
Supplement: Attachment — Submitted filename: comments-ellerman.pdf [file pone.0266800.s005.pdf]

Review Comments to the Author

I was glad to have the opportunity to review this manuscript, as risk communication research as a whole does need to be improved starting actually from the communication of risk assessment process and results, that to be effective, needs to be crafted according to several factors and this paper well shows this complexity. The very first step to have food risks appropriately perceived and managed – both by risk manages and consumers – is to translate the output of the risk assessment into relevant, understandable, reliable, clear and possibly "operational" information/instruction to face that risk.

Therefore, I warmly recommend the publication of this work; as well I encourage the authors to conduct additional research to fix the underlined criticalities, as stated in the Discussion session (line 406). This is an important result that emerges from this work.

In addition, this work (the risk profile tool) has the potential to be adopted by the wider community of risk communication practitioners, and serve as a tools for example to make comparisons between countries in terms of use, understanding and increasing of risk communication efficacy to both risk managers and consumers.

Before publication, I would suggest some little changes. The paper is well written but I think that some little improvements could be done

- Line 97: a general definition of risk profile should be given (what is it? What is it meant for? What content/information should it deliver? Who prepares a risk profile?...); I understand that is resembles the description of the BfR risk profile and that this can be inferred from the text, but it is better to provide the reader with a general / ideal one and this study helped to find the best working one so far. See for example section 3.3 in EFSA's Technical assistance in the field of risk communication https://efsa.onlinelibrary.wiley.com/doi/pdf/10.2903/j.efsa.2021.6574
- Line 176: how did you perform this task? Keywords used, websites/search engines searched, …
- Line 199: you mention here the "user-guide on how to complete the risk profile": did you produce it as an output of Step 4? I don't understand whether the "user-guide" is the risk profile template without information, simply the grid, or it is something different, e.g. a text that helps (guides) risk assessors to fill in the risk profile template with all the information needed?
- Line 311: step 3: which risk profile versions were discussed? Although your work is very detailed, it is difficult to seek for information through the main text and the supplementary materials, and the reader gets lost or does not easily remember each step of the methodology and the materials used at every given moment.
- Focus group results: did you consider creating a final table/figure to summarize focus group results to highlight common suggestions and discrepancies? I understand that the interview guides were different for each target audience, but the categorisation of results could help you draw a final map with major findings
