# Supplementary material for: Identifying content to improve risk assessment communications within the Risk Profile: Literature reviews and focus groups with expert and non-expert stakeholders
Source: PLoS One. 2022 Apr 11;17(4):e0266800. doi: 10.1371/journal.pone.0266800 (PMC9000125; doi:10.1371/journal.pone.0266800)
Supplement: Attachment — Submitted filename: Response to Reviewers.pdf [file pone.0266800.s006.pdf]

We thank the Editor Dr. Tim Mathes and the reviewers for the positive evaluation of our research, and for the many constructive suggestions to improve our manuscript. We have attempted to address all comments as we revised the manuscript. Our responses to the comments are provided in detail below.

1. Please review your reference list to ensure that it is complete and correct. If you have cited papers that have been retracted, please include the rationale for doing so in the manuscript text, or remove these references and replace them with relevant current references. Any changes to the reference list should be mentioned in the rebuttal letter that accompanies your revised manuscript. If you need to cite a retracted article, indicate the article's retracted status in the References list and also include a citation and full reference for the retraction notice.

We checked again the reference list and made sure all cited references are included.

2. Please ensure that your manuscript meets PLOS ONE's style requirements, including those for file naming. The PLOS ONE style templates can be found at

https://journals.plos.org/plosone/s/file?id=wjVg/PLOSOne_formatting_sample_main_body.pdf and

https://journals.plos.org/plosone/s/file?id=ba62/PLOSOne_formatting_sample_title_authors_affiliations.pdf.

We have checked our manuscript against these requirements.

3. We note that the grant information you provided in the 'Funding Information' and 'Financial Disclosure' sections do not match.

We apologise that there may have been a misunderstanding. The study did not receive official funding. The study was financed by in-house funds of the two study partners and the contributions were contractually agreed between the two project partners.

"The study received financial support from the German Federal Institute for Risk Assessment (BfR) and the Max Planck Institute for Human Development (MPIB). The funding agreement ensures the authors' independence in designing the studies, interpreting the data, writing, and publishing reports.

www.bfr.bund.de/en/home.html
www.mpib-berlin.mpg.de/en"

When you resubmit, please ensure that you provide the correct grant numbers for the awards you received for your study in the 'Funding Information' section.

Not applicable

4. In your Data Availability statement, you have not specified where the minimal data set underlying the results described in your manuscript can be found. PLOS defines a study's minimal data set as the underlying data used to reach the conclusions drawn in the manuscript and any additional data required to replicate the reported study findings in their entirety. All PLOS journals require that the minimal data set be made fully available. For more information about our data policy, please see http://journals.plos.org/plosone/s/data-availability.

Upon re-submitting your revised manuscript, please upload your study's minimal underlying data set as either Supporting Information files or to a stable, public repository and include the relevant URLs, DOIs, or accession numbers within your revised cover letter. For a list of acceptable repositories, please see http://journals.plos.org/plosone/s/data-availability#loc-recommended-repositories. Any potentially identifying patient information must be fully anonymized.

Important: If there are ethical or legal restrictions to sharing your data publicly, please explain these restrictions in detail. Please see our guidelines for more information on what we consider unacceptable restrictions to publicly sharing data: http://journals.plos.org/plosone/s/data-availability#loc-unacceptable-data-access-restrictions. Note that it is not acceptable for the authors to be the sole named individuals responsible for ensuring data access.

We will update your Data Availability statement to reflect the information you provide in your cover letter.

As stated in our previous submission, we are unable to share the transcripts from the focus group interviews as they contain potentially identifying or sensitive participant data. Participants were not informed or requested to allow for the data to be shared publicly. However, we have now uploaded the f4 analysis codebook from our qualitative analysis software to the Open Science Framework. The codebook details the coding of the transcripts and therefore represents the underlying data for our focus group analyses. We have modified the data availability statement to read:

"Relevant data are within the paper and its Supporting Information files. The data underlying the sub-study (focus group interviews) contain sensitive information and are protected by the Data Privacy Act. Thus, we are not able to share the transcripts from the focus group interviews. The study was approved by the Institutional Ethics Board of the Max Planck Institute for Human Development, Berlin, Germany. Interested researchers can contact the ethics committee of the Max Planck Institute for Human Development (Dr. Uwe Czienskowski, sciencec@mpibberlin.mpg.de).
A Codebook with details on the coding of the transcripts and number of text passages in the code per interview is available on the open science framework (https://osf.io/eqtd7/). "

5. We note that you have indicated that data from this study are available upon request. PLOS only allows data to be available upon request if there are legal or ethical restrictions on sharing data publicly. For more information on unacceptable data access restrictions, please see http://journals.plos.org/plosone/s/data-availability#loc-unacceptable-data-access-restrictions.

In your revised cover letter, please address the following prompts:

a) If there are ethical or legal restrictions on sharing a de-identified data set, please explain them in detail (e.g., data contain potentially sensitive information, data are owned by a third-party organization, etc.) and who has imposed them (e.g., an ethics committee). Please also provide contact information for a data access committee, ethics committee, or other institutional body to which data requests may be sent.

b) If there are no restrictions, please upload the minimal anonymized data set necessary to replicate your study findings as either Supporting Information files or to a stable, public repository and provide us with the relevant URLs, DOIs, or accession numbers. For a list of acceptable repositories, please see http://journals.plos.org/plosone/s/data-availability#loc-recommended-repositories.

We will update your Data Availability statement on your behalf to reflect the information you provide.

As described above, we cannot publish the focus group transcripts for data privacy reasons. We therefore provide a clear process and contact information for our institutional ethics board for readers who would like further information. We also now include the data from our coding analysis on the Open Science Framework.

Reviewer #1:

The paper addresses a specific case related to BfR's role and work in Germany in the continuous development of comprehensible risk communication. The background, as inferred by the paper, is that different target groups involved in the risk communication chain have different views on importance, content, and outcome of the communication.

In this paper qualitative focus group methodology has been used to gather in-depth insights into aspects of the Risk Profile that may improve risk messaging. As such qualitative methodology does not provide generalisable results or knowledge of how a topic is understood in the general or target population. However, by taking certain precautions in the recruitment of respondents, valuable input and insight can be provided.

The paper addresses these issues in a satisfactory way.

We thank the reviewer for his positive evaluation of our study and the contributions of our manuscript.

I have one question: Is the qualitative evaluation of the 2013 Risk Profile conducted in 2015 published somewhere?

The report on the BfR evaluation of the 2013 Risk Profile is an internal report and unfortunately is not intended to be published by the BfR.

Reviewer #2:

This is an interesting paper with the aim of improving the presentation methods for risk profiles on foods and consumer products as provided by risk assessment agencies. I believe this is an important area of development given the target audiences of these assessments and the increasing interest in science communication. The methods used are appropriate and generally well reported. It was a pleasure to review this manuscript. I have a few specific comments on the manuscript, which I hope are helpful.

We thank the reviewer for his positive evaluation of our study and the contributions of our manuscript.

General note:

Some parts of the manuscript seem unnecessarily lengthy in my opinion, e.g. the explanation of a

PICO in S1 (section 2.1) and of the search techniques (use of thesaurus, key words, Boolean Operators). I don't think it is necessary to describe standard review methods as long as it is reported in a transparent and reproducible way what was specifically done in the given review (e.g. reporting of the PICO elements, databases searched, search strategy etc.). I think it would make the paper more digestible to abbreviate such passages, but I understand that such choices can be deliberate depending on the audience.

We thank the reviewer for this suggestion. We have now shortened some of these standard review paragraphs to make the paper more digestible for the reader.

Background:

In line 87/88 you refer to "EFSA explains" factsheets targeted at consumer audiences. This seems contradictory to step 1 (lines 209/10), where you state that searches for risk communication strategies used by international agencies did not yield any applicable results. Furthermore, you mention that there were some exceptions to this (line 211). It would be interesting to know whether and how these were considered or – if not – why they were not deemed relevant. Lastly, were there any relevant differences between the 2013 BfR version of the Risk Profile and the adapted Swedish "risk thermometer" that may have been relevant to advancing the BfR tool?

We appreciate the reviewer highlighting these apparent contradictions in our explanation of the initial search. The 'EFSA explains' factsheets do directly target consumer audiences and focus on providing easy to understand explanations for certain consumer-relevant topics. While some of these factsheets include visual elements and are structured to answer key risk questions, the factsheets are highly tailored to the specific risk assessment topic and do not provide a standardized approach that can be applied across risk assessment topics. Indeed, their structure and included visuals can differ quite markedly across topics. For these reasons, the EFSA explains factsheets were not considered as an existing standardized approach to assist our development of a standardized tool.

We have revised the manuscript in the Introduction, when we first mention the EFSA explains factsheets to highlight this difference:

"At the European level, risk assessments that are considered to be of particular interest to the public are communicated in formats targeted at more general audiences, such as the European Food Safety Authority's "EFSA explains" Factsheets that develop tailored communications for specific topics (e.g., on caffeine, salmonella, or acrylamide in food; [5])"

We also include an explanation in the Method section where we describe the initial search method:

"The search revealed that almost all institutions used scientific articles or reports to publish their risk assessments. Further, none used a standardized approach to communicate results of individual risk assessments to lay audiences, with some exceptions. The European Food Safety Authority produces factsheets that are non-standardized, tailored communications for specific risk assessments of public interest [5] and general information on risk assessments or food safety topics in the form of infographics [6])."

We also added some details on risk thermometer from the Swedish National Food Agency (NFA):

"The risk thermometer compares food-related risks by combining probability, severity and uncertainty into a single metric that is visualized in the form of a thermometer. The purpose of the

tool is to enable direct comparisons of food risks according to this combined metric and does not provide an overview of the risk assessment topic in a format that is targeted at lay audiences."

In line 94, you mention formats to disseminate findings from systematic reviews to non-expert audiences such as plain language summaries. However, it appears that reference 9 and 10 refer to studies conducted with health professionals or researchers. This seems misleading and I believe that there are better suited references, e.g. doi:10.1016/j.jclinepi.2014.04.009.

We thank the reviewer for drawing our attention to the more suitable reference. We have now cited it in the relevant section of the manuscript.

Methods/Results:

Line 235: I suggest rephrasing this. As I understand, you searched systematic reviews and trials on the effects of different communication strategies, not "current best practice recommendations" (e.g. as provided here: 10.1136/bmjopen-2019-036348). Also check line 400 on this.

Thank you for your comment, we have rephrased these sentences:

"Specifically, we reviewed literature on communicating the following key questions" (line 235)
and
"Further, for most of the five risk characteristics, there were no high quality studies (randomized controlled trials) or reviews for communicating results about the severity of health impairments, dose-response thresholds, or the uncertainty or quality of evidence." (line 400)

Lines 247 ff: It should be mentioned here that not all the listed databases were searched for all key questions.

We have revised the sentence in line 249 to read:

"Owing to the different content within each topic, the databases were adjusted to the specific search such that the same set of databases were not used for all key questions. Specifically, for some searches, additional databases were employed to find relevant studies (e.g., the use of educational research databases to search for dose-response relationships."

In the main manuscript it would be useful to state whether the focus group members received the Risk Profile beforehand. I did not find this information.

Only some of the risk assessors and risk managers were aware of the 2013 Risk Profile or had worked with it previously. As such, at the beginning of the session, risk assessors were asked for their opinion on the 2013 Risk Profile and its individual characteristics, as well as for ideas on how to improve the profile after presentation (line 290). We added the information that the 2013 Risk Profile was presented to risk assessors at the beginning of the focus group interviews.

"After presentation of the 2013 Risk Profile participants were asked for their opinions on the profile (see Fig 1) and its individual characteristics." (line 304)

Participants from general population and risk managers were not shown the original 2013 Risk Profile but were instead shown two different prototypes of the risk profile (see Fig. 3) that were revised based on the literature review and feedback from the risk assessors interviews. These prototypes presented each key characteristic separately (i.e., participants did not receive a complete risk profile). In line 324 we wrote:

„Semi-structured focus group interviews were conducted with risk managers and members of the general public to elicit feedback on risk profile prototypes developed on the basis of Step (1) and (2), and to inform further revisions of the prototypes prior to user testing"

The final version of the Risk Profile on magnesium is an example with a verbal presentation of risks. It would be interesting to see an example where adverse effects are presented numerically.

Unfortunately, neither of the two risk assessment topics selected for the profile development contained numerical risks on adverse effects. These topics were chosen because they were everyday risks that would be relevant to the different user groups, and they presented different risk communication challenges across disciplines at the BfR (e.g., familiarity among consumers, communication of health based guidance value). Although we did not create a risk profile version that included numerical risks, there is extensive evidence from the risk communication literature on how to present numerical risks to facilitate understanding (e.g., absolute numbers or simple frequencies as opposed to relative risks). These recommendations are included in the user guide to inform how to present numbers within a risk profile (e.g., whenever possible, probability should be quantified with probabilities represented in absolute numbers: e.g., 3 out of 4 people who take more than [x mg] of [substance X] develop [endpoint]").

We have now included this point in our Discussion section relating to quantifying probabilities in risk assessments, and refer to the extensive literature on presenting numerical probabilities to guide the reader to best practice recommendations:

"Indeed, although we did not include a risk assessment topic that presented risks numerically, there is an extensive literature on how to present probabilities in formats that facilitate understanding (e.g., absolute numbers rather than relative risks; including information on base rates or reference groups; Bonner et al. 2021)."

Discussion:

The limitations section is relatively short and could address more issues. One limitation, for example, is that prototype V2 was not reassessed with risk managers and the members of the public. Thus, I am not sure the process is truly iterative. A second round could have highlighted additional information – for example (I am just thinking loudly here), how people feel about the presentation of the likelihood of occurrence for a low intake (vs. high intake or presentation of both).

The reviewer is correct that we did not reassess the V2 prototype with risk managers and citizens, only with risk assessors. Our intention was to test the complete profile in a following step with a randomized controlled trial with members from the general public. We have included this point in the limitations section of the Discussion.

"The V2 prototype was not assessed with risk managers or participants from the general public; only with risk assessors in the form of usability tests where only minor changes to the prototype V2 were made. Nevertheless, we are evaluating the effect of the newly developed final Risk Profile (2020 Risk Profile) on improving comprehension of risk assessment results in a randomized trial with members of the general public."

We also include a section in our Limitations to discuss the reviewer's point about certain presentations of risks not being addressed in the current study:

“Further, while we aimed to improve risk assessment communication over a broad range of topics, certain topics may not have been covered sufficiently. For instance, the topics we examined may not have been able to measure how people evaluate the probability of occurrence depending on the severity (mild vs. severe health consequences), the probability of contact (for instance, high consumption vs. low consumption) or differences in the quality of evidence (low uncertainty vs. high uncertainty). These questions warrant further research in future studies.”

Also, the interview guide provided in S3 seems ambitious for 1.5 to 2h focus groups. It would be interesting to reflect on this and whether there were topics that could not be addressed due to time constraints, especially among members of the public not familiar with the topic.

The reviewer is correct that the interview transcript included a lot of questions for the focus groups. However, despite time constraints, we managed to ask all our intended questions within the timeframe, and sought to facilitate the groups so that all participants were able to contribute throughout the interviews. Nevertheless, given the many questions we had scheduled, it may have been the case that additional time dedicated to each question would have benefited the diversity of content we elicited from the group.

One important aspect of communication is the distribution of the information. This receives little attention in the manuscript. It would be interesting to discuss this aspect. To name one example, social media and mobile devices are major channels for infographics, but have specific requirements (size constraints, limited attention, “mobile first” etc.). Thus, the Risk Profiles would likely need to be modified for such purposes. The discussion provides an opportunity to highlight this limitation and opportunity for future work.

We thank the reviewer for pointing this out. The results of the risk assessments are published on the BfR website and serve as a basis for risk managers to make decisions or to communicate with consumers. In future, the risk profile could also be incorporated into direct communication to consumers (e.g., via the consumer protection centres’ website, facebook or twitter) or in various media reports dealing with the safety of certain foods or risks of consumers. We added this point for future research in the conclusion section.

Supplement 1:

The numbers in Figure 1 deviate from the numbers in the preceding text (e.g. figure 1 reports 4 additional records identified from gray literature, whereas the text passage mentions three records identified through Google Scholar, 406 at ti/ab stage in figure vs. 405 in text and so on). It also seems odd that there are duplicates, even though only one database was searched. Have you checked that this was not an error from the automated deduplication in EndNote?

We thank the reviewer for their attention to detail and picking up on these minor discrepancies in the numbers reported in our Supplementary material. We have rechecked our Endnote libraries and detected two errors due to the manual copying of studies during the screening process in Endnote: one study was excluded during the full text screening process but was inadvertently moved to the folder intended for papers excluded during title abstract screening; one study was erroneously included in triplicate). We have rechecked the remaining classifications to ensure no further errors in the Endnote classifications occurred.

There also seem to be errors in the other flow charts in S1 (e.g. page 15: 161, 66, 76 and 93 records equate to 396, while the figure reports 391 initial records). Please make sure the numbers throughout the manuscript and supplements are correct and consistent.

As stated above, we have rechecked the remaining Endnote classifications and flowcharts to ensure no further errors or inconsistencies occur in the manuscript and Supplementary Material. We have made the relevant corrections.

Minor:

Line 90: should it say "to health"?
Line 92: the semicolon after Cochrane Collaboration seems to be superfluous
Line 137: I think the comma after "managers" is not needed
Line 175: "initial first step" seems tautologic
Line 225: consider removing "all"

We have made each of the above mentioned suggested changes.

References: Please check the references for correct citation style. There seem to be some errors here, e.g. reference 7 (publisher missing) and 14 (first names of authors written out, "and" between authors).

We thank the reviewer for the note. We have corrected it.

Reviewer #3:

I was glad to have the opportunity to review this manuscript, as risk communication research as a whole does need to be improved starting actually from the communication of risk assessment process and results, that to be effective, needs to be crafted according to several factors and this paper well shows this complexity. The very first step to have food risks appropriately perceived and managed – both by risk manages and consumers – is to translate the output of the risk assessment into relevant, understandable, reliable, clear and possibly "operational" information/instruction to face that risk.
Therefore, I warmly recommend the publication of this work; as well I encourage the authors to conduct additional research to fix the underlined criticalities, as stated in the Discussion session (line 406). This is an important result that emerges from this work.

We thank the reviewer for the positive evaluation of our research. We are working towards further research on improving risk communication.

In addition, this work (the risk profile tool) has the potential to be adopted by the wider community of risk communication practitioners, and serve as a tools for example to make comparisons between countries in terms of use, understanding and increasing of risk communication efficacy to both risk managers and consumers.
Before publication, I would suggest some little changes. The paper is well written but I think that some little improvements could be done
- Line 97: a general definition of risk profile should be given (what is it? What is it meant for? What content/information should it deliver? Who prepares a risk profile?...); I understand that is resembles the description of the BfR risk profile and that this can be inferred from the text, but it is better to provide the reader with a general / ideal one and this study helped to find the best working one so far. See for example section 3.3 in EFSA's Technical assistance in the field of risk communication https://efsa.onlinelibrary.wiley.com/doi/pdf/10.2903/j.efsa.2021.6574

We added some more details on the risk profile in line 107.

“Thus the Risk Profiles present a summary of a risk assessment in a format that makes the key criteria comprehensible at a glance. The Risk Profiles are developed at the time of the risk assessment in close collaboration with risk assessors and the communications department.

- Line 176: how did you perform this task? Keywords used, websites/search engines searched, …

We have added more information on how we performed this task to line 212).

“We searched the BfR website on European and international co-operations (www.bfr.bund.de/en/european_and_international_co_operations-10361.html) as well as search engines (e.g., Google) for risk assessment or risk communication institutions worldwide. In addition, experts from risk communication department of the BfR, who were involved in the 2013 Risk Profile development, were asked to share any other risk communication tools or visualization methods they were aware of.”

- Line 199: you mention here the “user-guide on how to complete the risk profile”: did you produce it as an output of Step 4? I don’t understand whether the “user-guide” is the risk profile template without information, simply the grid, or it is something different, e.g. a text that helps (guides) risk assessors to fill in the risk profile template with all the information needed?

The user guide was developed on the basis of the final risk profile (2020 Risk Profile). It serves to provide risk managers with guidance and detailed instructions on how to complete each section of the risk profile.

We added some details on the user guide to the methods and results section of step 4 (usability test and guide development).

Method section: "The user guide was drafted based on the V2 prototype and aimed to provide risk assessors with guidance on how to complete the risk profile (e.g., what information to provide in each section; best practices for presenting numerical risks etc). It was developed prior to the usability tests to assist risk assessors in completing a Risk Profile based on one of their recent risk assessment topics, and would be revised based on feedback from risk assessors during the usability tests.”

Results section: “Further, the draft user guide was developed in collaboration with risk assessors during the usability test. The purpose of the user guide was to provide risk managers with detailed instructions on how to complete each section of the risk profile, including guidance on what information to include and recommendations about what formats to present information to facilitate interpretation (e.g., to include numerical probabilities, if available, in absolute rather than relative numbers). These recommendations were also informed based on findings from the literature reviews (e.g., see Table 1). The draft user guide is currently being revised in coordination with working groups within the BfR.”

- Line 311: step 3: which risk profile versions were discussed? Although your work is very detailed, it is difficult to seek for information through the main text and the supplementary materials, and the reader gets lost or does not easily remember each step of the methodology and the materials used at every given moment.

We have revised Figure 2 to more clearly identify the various stages involved in the profile development, the insights gained at each stage, and the specific risk profile prototypes developed and/or shown to participants at each point in time. We have also now included more references to

Figure 2 throughout the text to remind the reader of each stage. We hope this helps improve the reader's orientation about the development process.

- Focus group results: did you consider creating a final table/figure to summarize focus group results to highlight common suggestions and discrepancies? I understand that the interview guides were different for each target audience, but the categorisation of results could help you draw a final map with major findings

As the reviewer suggests, as the results of focus group interviews were used to inform subsequent interviews and to develop and revise different prototypes, we did not summarise the results in a single table across all focus group studies. We have, however, attempted to summarise the changes to the prototype examples V1 from focus groups with risk managers and the general public in Table 3, as the interview guides and aim of focus group interviews were similar.
